# Expression and Refolding of the Plant Chitinase From *Drosera capensis* for Applications as a Sustainable and Integrated Pest Management

**DOI:** 10.3389/fbioe.2021.728501

**Published:** 2021-09-21

**Authors:** Igor G. Sinelnikov, Niklas E. Siedhoff, Andrey M. Chulkin, Ivan N. Zorov, Ulrich Schwaneberg, Mehdi D. Davari, Olga A. Sinitsyna, Larisa A. Shcherbakova, Arkady P. Sinitsyn, Aleksandra M. Rozhkova

**Affiliations:** ^1^ Federal Research Centre Fundamentals of Biotechnology, Russian Academy of Sciences, Moscow, Russia; ^2^ Institute of Biotechnology, RWTH Aachen University, Aachen, Germany; ^3^ Department of Chemistry, M.V. Lomonosov Moscow State University, Moscow, Russia; ^4^ DWI-Leibniz Institute for Interactive Materials, Aachen, Germany; ^5^ Department of Bioorganic Chemistry, Leibniz Institute of Plant Biochemistry, Halle, Germany; ^6^ All-Russian Research Institute of Phytopathology, Moscow, Russia

**Keywords:** Drosera capensis, plant chitinase (GH19), refolding, site-directed mutagenesis, molecular dynamics

## Abstract

Recently, the study of chitinases has become an important target of numerous research projects due to their potential for applications, such as biocontrol pest agents. Plant chitinases from carnivorous plants of the genus *Drosera* are most aggressive against a wide range of phytopathogens. However, low solubility or insolubility of the target protein hampered application of chitinases as biofungicides. To obtain plant chitinase from carnivorous plants of the genus *Drosera* in soluble form in *E.coli* expression strains, three different approaches including dialysis, rapid dilution, and refolding on Ni-NTA agarose to renaturation were tested. The developed « Rapid dilution » protocol with renaturation buffer supplemented by 10% glycerol and 2M arginine in combination with the redox pair of reduced/oxidized glutathione, increased the yield of active soluble protein to 9.5 mg per 1 g of wet biomass. A structure-based removal of free cysteines in the core domain based on homology modeling of the structure was carried out in order to improve the soluble of chitinase. One improved chitinase variant (C191A/C231S/C286T) was identified which shows improved expression and solubility in *E. coli* expression systems compared to wild type. Computational analyzes of the wild-type and the improved variant revealed overall higher fluctuations of the structure while maintaining a global protein stability. It was shown that free cysteines on the surface of the protein globule which are not involved in the formation of inner disulfide bonds contribute to the insolubility of chitinase from *Drosera capensis*. The functional characteristics showed that chitinase exhibits high activity against colloidal chitin (360 units/g) and high fungicidal properties of recombinant chitinases against *Parastagonospora nodorum.* Latter highlights the application of chitinase from *D. capensis* as a promising enzyme for the control of fungal pathogens in agriculture.

## Introduction

Chitin is the second most common natural polymer, predominantly consisting of b-1,4 linked molecules of N-acetyl-D-glucosamine, which forms the basis of the cell walls of fungi, the exoskeleton of insects and shells of crustaceans. Chitinases (EC 3.2.1.14) divided into two groups: exochitinases and endochitinases. Endochitinases cut the chitin at random sites internally and form soluble low molecular mass multimers of N-acetylglucosamine with degree of polymerization from 2 to 6. Exochitinases, in turn, are further divided into two groups: chitobiosidases and 1,4-β-glucosaminidases. Chitobiosidases catalyze the subsequent release of di-acetylchitobiose beginning at the non-reducing of the chitin fibrils. The 1,4-β-glucosaminidases cleave the oligomeric products of endochitinase and chitobiosidases to produce of glucosamine. Endochitinases are divided into two main glycoside hydrolases families 18 (GH18) and 19 (GH19). The endochitinases of GH18 are characteristic enzymes for a wide range of organisms: bacteria, fungi, insects, arthropods, and mammals. These enzymes play a key role in the development of the cell wall of organisms using chitin as a structural component, as well as these are the main enzymes involved in the degradation of chitin in nature (Javed et al., 2013).

Endochitinases related to GH19 are predominantly plant proteins and have a number of unique properties: they are able to efficiently hydrolyze crystalline chitin, are involved in the immune response of plants, and, are an effective cryoprotectant (Fernandez-Caballero et al., 2009). The significant fungicidal activity of chitinase GH19 was previously reported in many studies (Broekaert et al., 1992; Büchter et al., 1997).

Chitinases GH19 are able to bind to chitin polymers and catalyze its hydrolysis, also they are responsible for most of the chitinolytic activity in plants. Chitinases GH19 are divided into four classes (Javed et al., 2013). The most complete understanding of the structure of chitinases is formed by class I chitinases, which are characterized by four sequential domains such as a hydrophobic N-terminal signal peptide; a conservative cysteine-rich hevein-like domain (also called chitin-binding domain), a highly variable proline-rich linker and a catalytic domain (Taira, 2010). The structure of chitin-binding domains (ChBD) is very conservative, but their presence in the enzyme is not obligatory (Iseli et al., 1993; Price et al., 2015). The ChBD is docked to a catalytic domain with a highly variable proline-rich linker (Rafiq et al., 2018), that varies in sequence as well as in length for different chitinases.

Chitinases GH19 from plants are the proteins associated with pathogenesis, their function allows plants to resist the action of phytopathogens, however, some genera of carnivorous plants, *Dionaea*, *Drosera*, *Nepenthes* and *Triphyophyllum* in particular, have adapted these proteins for active hunting and degradation of insect chitin (Chase et al., 2009). It is hypothesized that divergence of chitinase genes could occur during the evolution of carnivorous plants, which led to the appearance of chitinolytic enzymes adapted for carnivory (Malik, 2019).

The pioneering study of the carnivorous plants chitinases reveal the duality of the properties of chitinases from carnivorous *Nepenthes* (Eilenberg et al., 2006). Expression of chitinase from *Nepenthes* was induced by colloidal chitin by a mechanism similar to the plant response to pathogen infection. However, it was shown that the induction of chitinase expression is necessary mainly for plant nutrition, but does not exclude their protective role. First chitinase from the sundew *Drosera rotondofula* was isolated and described in 2016, however, the authors described the significant difficulties in the preparation of a soluble form of the protein in the *E. coli* expression system (Rajinex et al., 2016). Using refolding methods, a soluble form of the enzyme was obtained and сhitinase showed the highest activity towards glycol chitin (92.20 ± 6.74 U/mg) and chitin powder (85.41 ± 4.1 U/mg). Quantitative *in vitro* assays showed growth suppression of *Fusarium poae* (40%), *Trichoderma viride* (43.8%), and *Alternaria solani* (52.6%) but not *Rhizoctonia solani* (Rajninec et al., 2020). Successful expression of chitinases from other plants were performed (Andersen et al., 1997; Kirubakaran and Sakthivel, 2007; Tanaka et al., 2017).

The prospect of using chitinases as protective agents against pathogenic organisms, such as fungi and insects, is of considerable applied interest, since it makes it possible to switch from the use of frequently mutagenic pesticides to biocontrol of the spread of phytopathogens based on the chitin-degrading function of chitinases (Kumar et al., 2018).The main limiting factor in the global integration of family 19 chitinase as an alternative (or as a supplement) to chemical plant protection against phytopathogens is the difficulty of obtaining a large quantity of inexpensive recombinant protein.

This is because bacterial systems are poorly adapted for the expression of cysteine-rich proteins (Baneyx, 1999). The correct formation of disulfide bonds is a “bottleneck” of bacterial expression. Moreover, there is compelling evidence that the presence of free cysteines on the surface of a protein globule can reduce the stability of the protein and lead to aggregation (Robinson and Sauer, 1998; Schmiedl et al., 2000). A high cysteine content is typical for the chitinase 19 family based on the analysis of the primary sequences of these proteins. Using the example of bacterial chitinases of family 19 with simplified structure (Itoh et al., 2002; Udaya Prakash et al., 2010), it was shown that modeling of the protein globule to remove the surface-free cysteines can eliminate molecular effects causing solubility problems. The study of new chitinases and the development of technology for their efficient production is an important task for further agriculture applications (Oyeleye and Normi, 2018).

In the work, the problem of efficient expression and refolding of chitinase from *D. capensis* as a sustainable solution for integrated pest management was solved. Refolding scheme for generation soluble full-length and truncated chitinase forms from inclusion bodies was developed. The *in silico* modeling to choose free cysteines on the surface of the protein globule was employed. The variants of mutant chitinase were obtained using site-directed mutagenesis. The improving of solubility mutant form (C191A/C231S/C286T) was confirmed. Molecular dynamics (MD) simulation was further used to explain the improved solubility at molecular level. Finally, the functional characteristics of Chit19 by evaluating their catalytic properties and fungicidal properties were characterized.

## Material and Methods

### Bacterial Strains

DNA manipulations were performed in *E. coli* DH5α (*hsdR17(r*_*K*_^*−*^ *, m*_*K*_^*+*^) *supE44 thi-1 recA1 gyrA* (*Nal* ^*r*^) *relA1 Δ*(*lacZYA-argF*)_U169_ (*m80lacZΔM15*) (Thermo Fisher Scientific Inc., Waltman, MA, United States). Recombinant Chit19 was expressed in *E. coli BL21(DE3) Arctic Express* (*F* ^*−*^ *ompT hsdS* (*rB–mB* ^*–*^) *dcm* ^*+*^ *Tet* ^*r*^ *gal* λ(*DE3*) *endA Hte* (*cpn10 cpn60 Gent* ^*r*^ ] (Agilent, Santa Clara, CA, United States) and *E. coli BL21(DE3) Rosetta-gammi 2* (Δ(*ara-leu*)7,697 Δ*lacX74 ΔphoA PvuII phoR araD139 ahpC galE galK rpsL* (*DE3*) F′(*lac* ^*+*^ *lacIq pro*) *gor522:Tn10 trxB* pRARE2 (*Cam* ^*R*^ *, Str* ^*R*^ *, Tet* ^*R*^)) (Sigma-Aldrich, St. Louis, MO, United States) strains.

### Isolation and Cloning of the *сhit19* Gene

The CTAB protocol (Allen et al., 2006) has been adapted for the isolation of genetic material from *D. capensis*. Briefly, leaves (100 mg) were ground in liquid nitrogen, the resulting powder was transferred to preheated lysis buffer (2%) cetriltrimethylammonium bromide (CTAB); 1.5 M NaCl; 2% β-mercaptoethanol; 4% Polyvinylpyrrolidone (PVP) 15k; 100 mM Tris-HCl; 10 mM Ethylenediaminetetraacetic acid (EDTA), pH 8.0, 60°C) and incubated for 10 min. The resulting solution was centrifuged (14 ,000 g, 10 min, 4°C). The supernatant was transferred to a clean tube, an equal volume of chloroform was added, stirred by shaking, and then centrifuged (14 ,000 g, 100 min, 4°C). Three volumes of chilled ethanol and 0.1 volume of 3M sodium acetate (pH 4.5) were added to the supernatant in succession. The solution was incubated for 2 h at –80°C and centrifuged (14 ,000 g, 30 min, 4°C). The supernatant was removed, and the pellet was dissolved in 200 μl of ddH_2_O. 10M LiCl was added to the solution to a final concentration of 2.5M for RNA from DNA separation, followed by incubation for 2 h at −80°C and centrifugation (14 ,000 g, 10 min, 4°C). The resulting RNA pellet was washed twice with 70% ethanol, dissolved in 50 μl of ddH_2_O and treated with DNase (Thermo Fisher Scientific Inc., Waltman, MA, United States) in accordance with the manufacturer’s recommendations. The resulting purified RNA solution was used to generate the cDNA library. The first cDNA strand was synthesized using the Maxima H Minus First Strand cDNA Synthesis Kit (Thermo Fisher Scientific Inc., Waltman, MA, United States) according to the manufacturer’s instructions. The cDNA library was synthesized from 1 µg of the total RNA using a (dT_18_) primer. Degenerate primers ChitFS and ChitRS (Supplementary Table S1) were synthesized to amplify the primary *chit19* gene fragment encoding chitinase from *D. capensis* using a strategy based on the homology of conserved regions. The flanking regions of the gene were restored by TAIL-PCR (Jia et al., 2017).

### Design of the Recombinant Expression Plasmid

Both forms of chitinases (with (Chit19) and without (Chit19ΔChBD)) were cloned into pET28a expression vector (containing the 6x-His-Tag) by the *Nde*I/*EcoR*I restriction pair sites. The resulting plasmids, pETChit19 and pETChit19ΔChBD (Supplementary Figure S1), were sequenced into both directions by Sanger method (Sanger et al., 1980), using primers from Supplementary Table S1.

### Chitinase Expression and Isolation of Inclusion Bodies

The recombinant pETChit19 and pETChit19ΔChBD plasmids were transformed into *E. coli BL21(DE3) Arctic Express* and *E. coli BL21(DE3) Rosetta-gammi 2* according to the standard protocol. In brief, the transformants were grown at 37°C in 100 ml of TB culture medium supplemented by 50 μg/ml Kanamycin (Sigma-Aldrich, St. Louis, MO, United States) and 250 rpm to an OD_600_ = 0.3. Then 0.3 mM isopropil-β-D-tiogalactopiranoside (IPTG) (Sigma-Aldrich, St. Louis, MO, United States) was added and growth was continued for 24 h at 11°С in case of *Arctic Express* and 24 h at 18°С *Rosetta-gammi 2*. At the end of cultivation, the cells were resuspended in 10 ml of lysis buffer (50 mM Tris-HCl, 100 mM NaCl, 5 mM Dithiothreitol (DTT), 1 mM Phenylmethylsulfonyl fluoride (PMSF), pH 7.5) and lysed by sonication on ice (5 times for 30 s). The bacterial lysates were centrifuged for 60 min at 4°С (15,000 rpm), supernatants and precipitates were separated and identified by SDS-PAGE. The precipitate was washed with 5 ml of wash buffer (50 mM Tris-HCl, 100 mM KCl, and 2M Urea, pH 8.5) five times to maximize the removal of *E. coli* proteins. Inclusion bodies were pelleted for 15 min at 21,400 g, the supernatant was removed. The precipitate was dissolved in 5 ml of denaturing buffer (50 mM Tris-HCl, pH 8.5, 100 mM KCl, 8M urea, 1 mM EDTA and 20 mM DTT) over 2 h at 40°C. The solution was centrifuged for 15 min at 15,000 rpm. Protein concentration in the supernatant was determined by absorption at 280 nm using NanoDrop Lite (Thermo Fisher Scientific Inc., Waltman, MA, United States) and used for further work.

### Refolding Procedure

The key points of refolding optimization were two main directions: 1) the composition of the refolding buffer and 2) the method of denaturing agent removal. Chemical additives are capable of replacing protein chaperones to a large extent for the correct folding of biomolecules (Yamaguchi et al., 2013). In this work, the contribution of various additives to the refolding buffer was evaluated. The main refolding buffer consisted of 50 mM Tris-HCl, pH 8.5 and 200 mM NaCl, and was supplemented with sucrose, proline, arginine and glycerol in various ratios. The choice of the redox pair has a significant impact on the rate of reassembly of the disulfide bond and the final yield of the soluble form of the protein. In our work we have used: cystine/cysteine, oxidized glutathione (GSSG)/reduced glutathione (GSH) and 2-mercaptoethanol additives. The use of detergents can also reduce protein aggregation, that’s why the effects of Triton X-100, Twin 20 and sodium lauryl sarcosyl supplementation have been tested (Zardeneta and Horowitz, 1994). The efficiency of refolding was assessed by the release of the soluble form of the enzyme and its activity. First, the effect of individual additives was assessed, after which the synergism was assessed using several of the most effective of them. The optimal conditions for removing the chaotropic agent from the denatured chitinase solution were determined after selecting the optimal buffer for refolding.

### Dialysis

The gradual removal of the chaotropic agent by dialysis is a classic technique that allows the chaotropic agent to be gradually removed from the medium, which enables proteins to restore their native structure. One milliliter of chitinase solution (2 mg/ml) in a denaturing buffer (8 M urea 50 mM Tris-HСl (pH 8.0), 100 mM NaCl) was dialyzed against the denaturing buffer, in which the urea concentration was gradually reduced every 2 h (urea concentration 6, 4, 2, 1, and 0.5 M, respectively) at 4°C. Thereafter, dialysis was performed for 24 h against urea-free refolding buffer. the resulting solution was separated from the sediment by centrifugation at 14,500 rpm and re-dialyzed against 20 mM Na-acetate buffer pH 5 containing 5% glycerol for 24 h. SnakeSkin^™^ Dialysis Tubing (10K MWCO, Thermo Fisher Scientific Inc., Waltman, MA, United States) were used for dialysis and refolding.

### Rapid Dilution

Rapid “shock” dilution of protein in refolding buffer in a ratio of 1–10 is preferable to gradual dialysis in some cases (Wang et al., 2017). One milliliter of chitinase solution in a denaturing buffer (2 mg/ml) was cooled to 4°C and quickly added to the cooled refolding buffer with vigorous stirring. The mixture was incubated for 24 h, separated from the sediment by centrifugation at 14,000 *g* for 15 min, the supernatant was dialyzed for 24 h against 20 mM Na-acetate buffer, pH 5.0, containing 5% glycerol.

### Refolding on Ni Sepharose

Renaturation on Ni-modified affinity carriers is a well-known technique that is often used to obtain soluble forms of the protein. Immobilization of denatured protein molecules on a carrier physically isolates the molecules from each other and minimizes intermolecular interactions during the folding process (Jopcik et al., 2017). 1 ml of Ni Sepharose^®^ Excel (GE Healthcare, Chicago, IN, United States) was added to 1 ml of chitinase solution (protein concentration 2 mg/ml) in a denaturing buffer and gently stirred for 30 min at 4°C. The adsorbed protein was washed with a denaturing urea gradient buffer (6, 4, 2, 1, and 0.5 M) and eluted from Ni Sepharose with an elution buffer (50 mM Tris-HCl (pH 8.0), 300 mM NaCl, 250 mM imidazole) gradient. The fractions containing the target protein were pooled. The resulting solution was centrifuged at 14,000 g and dialyzed for 24 h against 20 mM Na-acetate buffer, pH 5.0 containing 5% glycerol.

### Purification, MALDI-TOF Mass Spectrometry

The recombinant proteins were purified by HisTrap^™^ Excel 5 ml (GE Healthcare, Chicago, IN, United States) column, connected to the AKTA Purifier system (GE Healthcare, Chicago, IN, United States). The purity and mass of target proteins were assessed by SDS-PAGE (Bio-Rad Laboratories, Inc., Hercules, CA, United States). The in-gel tryptic digestion of protein bands after the SDS-PAGE was carried out essentially as described by Shevchenko (Shevchenko et al., 2007). Trypsin Gold (Promega, Madison, WI, United States, modified, 5 μg/ml) in 50 mM NH_4_HCO_3_ was used for a protein digestion. The resulting peptides were extracted from the gel with 20% aqueous acetonitrile containing 0.1% trifluoroacetic acid, and MALDI-TOF mass spectrometric analysis was performed using UltrafleXtreme TOF/TOF mass spectrometer (Bruker Daltonics GmbH, Bremen, Germany). Enzymes were identified by peptide fingerprint using the Mascot server (http://www.matrixscience.com/).

### Computational Analysis

Homology modeling of the Chit19 structure was performed using the YASARA software package for related sequence and template identification, modeling, and validation (Supplementary Figures S2 and S3; for computational details we generally refer to the SI). For modeling, the signal peptide sequence was predicted using the Signal-P 5.0 web server and was removed from the complete Chit19 sequence (Almagro Armenteros et al., 2019). Alignment-based sequence conservation analysis was performed to identify conserved and potentially evolutionary important residues using the webserver-based software tools ConSurf (Ashkenazy et al., 2010, 2016), BLAST (Altschul et al., 1997, 2005), WebLogo 3 (Crooks et al., 2004), and the evolutionary trace webserver (Wilkins et al., 2012) (Supplementary Figures S4–S6). Chit19 was examined regarding potential engineering prospects of free cysteines of the core domain for improving solubility based on the established homology model and evolutionary conservation analysis on a specific alignment of the Chit19 sequence to six homologous chitinases. Using the homology model as template, the identified free cysteines were substituted *in silico* to conserved or rationally derived amino acids and model variants were designed and analyzed regarding stability using the FoldX method (Schymkowitz et al., 2005). Both established models (Chit19 wild-type and free cysteine substituted variant) were analyzed regarding their flexibility of the 3D-structure studying each variant’s dynamics. Therefore, MD simulations (three independent runs for 100 ns) were performed to elucidate at molecular level the flexibility of the different regions of Chit19 and the impact of free cysteine substitutions on the dynamics on the Chit19 variant (for detailed description of the MD setup we refer the reader to the **SI**).

### Site-Directed Mutagenesis

**FIGURE 1:**
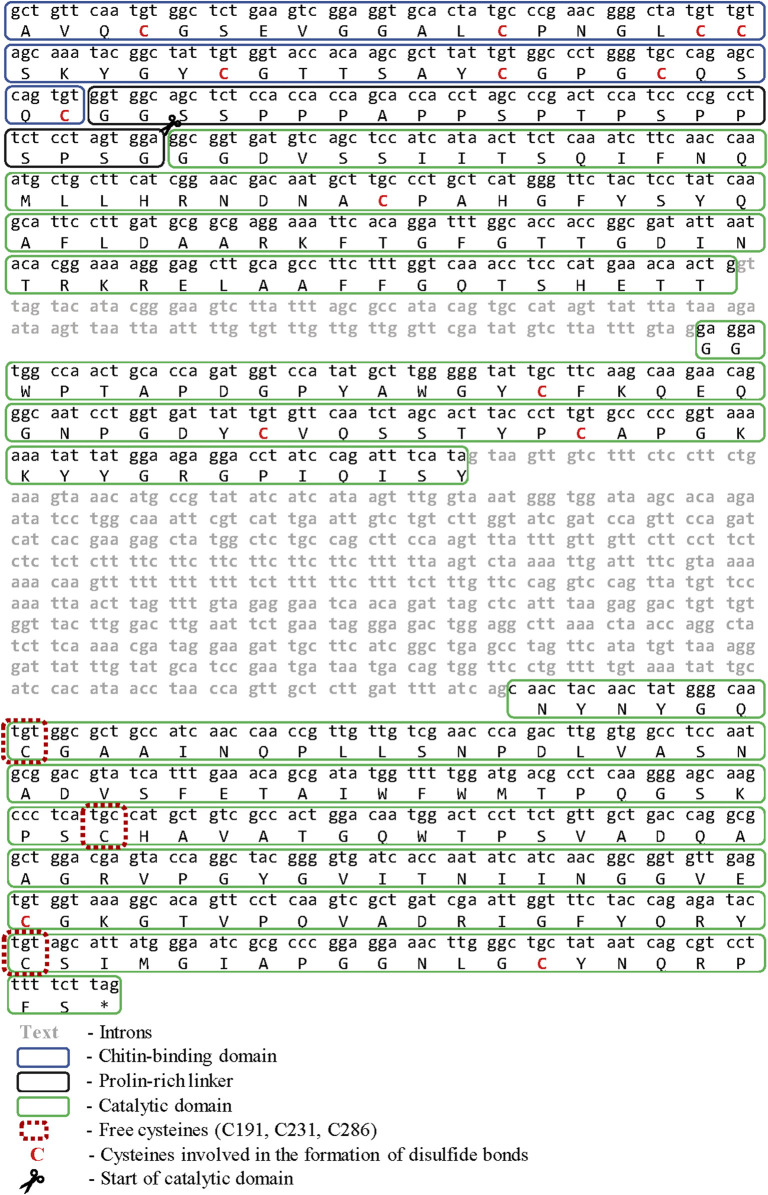
**-** Nucleotide and amino acid sequence Chit19.

Quick-change protocol was used to create mutant cysteine chitinases (Braman et al., 1996). Briefly, 40 ng of plasmid was added to 50 µl PCR mixture and amplified with Phusion ™ High-Fidelity DNA Polymerase (Thermo Fisher Scientific Inc., Waltman, MA, United States) and primer pairs (Supplementary Table S1, Figure 1). 10 units of DpnI restrictase was added to the reaction mixture before transformation and incubated for 2 h at 37°C. 5 μl of the resulting reaction mixture was used for *E. coli* transformation.

Mutant plasmids were obtained for both Chit19 and Chit19ΔChBD. Plasmid constructs were named pET_ChitC191A, pET_ChitC231S and pET_ChitC286T (see SI).

### Chitinase Activity Assay

The activity based on chitin and chitosan was determined by spectrophotometric detection of the N-acetylglucosamine released during the hydrolysis. A Na-acetate buffer (200 μl), pH 5.0, and 100 μl of the enzyme preparation (with the concentration of 1 mg/ml) were added to 200 μl of 2% colloidal or crystalline chitin suspension, the mixture was incubated at 55°C for 30 min. 1,000 μl of a staining solution (0.5 g/L potassium hexacyanoferrate (III) in 0.5 M Na_2_CO_3_) was added, the mixture was incubated for 15 min at 100 °C, centrifuged, and the absorbance was measured at λ = 410 nm using Cary 60 UV-Vis spectrophotometer (Agilent, Santa Clara, CA, United States). One unit of chitinase activity was defined as capable of releasing reducing ends corresponding to 1 µg of GlcNAc per minute. The analysis of chitosanase activity was carried out according to a similar protocol, but chitosan 140 kDa was used as a substrate. The analyzes were performed in triplicate.

### Antifungal Activity Assay

Antifungal effect of Chit19 was demonstrated against *Parastagonospora nodorum*, a pathogen causing leaf/glume blotch of wheat, using a detached leaf test detailed previously. (Shagdarova et al., 2018). Briefly, leaf sections (8 cm long) cut from central parts of detached wheat leaves (10 leaves per treatment) and placed in Petri dish atop of 1% water agar supplemented with benzimidazole (0.004%). *P. nodorum* spores were suspended in a portion of daily fresh 20 mM Na-acetate buffer, pH 5 buffer to a concentration of 2 × 10^6^ conidia/ml. Prior to inoculation of leaf sections, 1 ml of the suspension was mixed with 1 ml of the aforementioned freeze-dried preparation of the purified and refolded enzyme dissolved in the same buffer to a concentration of 0.5 mg/ml. Resulting mix contained Chit19 and fungal spores at the final concentrations of 1 × 10^6^ conidia/ml and 0.75 mg/ml, respectively. After 1-h incubation at 20–22°C, aliquots (10 µl) of the mix were dropped on upper (distal) parts of the leaf sections. In parallel, drops (10 µl) of conidial suspensions (10^6^ conidia/ml) in the buffer were applied to lower (basal) parts of the same leaf sections (control). An additional plate was prepared to observe disease on non-treated leaves, in which both distal and basal locations on each of 10 leaf sections were inoculated with pathogenic conidia in sterilized distilled water. Disease symptoms were assessed on a five-point scale 4 days after inoculation.

## Result and Discussion

The results section is divided into six parts. The first part describes cloning of the full-length gene of chitinase 19th family of the *D. capensis* and analysis of the possibility of recombinant expression in *E. coli* cells of the full-length form and truncated form (with the chitin-binding domain removed). The second part was devoted to the development of an optimal strategy for restoring the enzyme form from inclusion bodies. A comprehensive analysis of the methods for removing the denaturing agent to yield recombinant chitinase during refolding was carried out. At the third stage, an assessment of the effectiveness of various chemical additives on the process of refolding was studied. The fourth part was comprises creating and evaluating the structure of the Chit19 model and *in silico* design of free improved variants. This model has been proposed for the putative catalytic residues and positions of cysteine residues. In the fifth part, *in silico* guided site-directed mutagenesis was used to generate a form of chitinase with the replacement of free cysteines. In addition, molecular insight into improved chitinase solubility based on molecular dynamics modeling is provided. In the final part, the functional characteristics of all chitinase variants were explained in detail.

### Cloning and Isolation of *chit19* Gene

Fragment of the chit19 gene, 835 bp length was cloned using degenerate primers (Sinelnikov et al., 2020). Several rounds of TAIL-PCR were performed for the restoration of the terminal regions of the gene. Sequencing of the obtained PCR products made it possible to restore the structure of a 2,443 bp DNA fragment. Exon-intron structure analysis of the fragment using the AUGUSTUS server (Stanke and Waack, 2003) allowed to confidently determine one open reading frame, three exons, and two introns. The total gene length was 1724 bp and the length of the coding sequence was 978 bp (Figure 1). Full sequences of chitinase from *D. capensis* (Chit19) was submitted to the GenBank databases under accession number MK093978. In general, the presence of two to four introns is typical for plant chitinases. Chitinases from carnivorous plants have an identical gene structure: chitinases from *D. rotundifolia* (Jopcik et al., 2017) and *Nepenthes khasiana* (Eilenberg et al., 2006), which indicates a general homology of the origin of these chitinases.

### Expression Strategies

The amino acid sequence of full-length chitinase without native signal peptide from *D. capensis* is shown on Figure 1, disulfide bonds and free cysteines are marked red. The chitin-binding domain of chitinase GH19 (1–42 aa) has a high homology with the chitin-binding domains of chitinases from other plants (identity >90%). It has eight conserved cysteines and two tyrosine residues in its structure, which are involved in the binding to chitin (Price et al., 2015). The presence of a chitin-binding domain is not critical for the chitinase activity of chitinases. It can be assumed that the cysteine-rich chitin-binding domain will affect the expression and folding of the catalytic moiety of chitinase in the *E. coli* expression system, especially taking into account the structure of family 19 chitinases isolated from *Streptomises greseus* bacteria. Bacterial chitinase has six cysteines in its structure and several deletions, which are not observed in plant chitinases (Kezuka et al., 2006; Udaya Prakash et al., 2010).

**FIGURE 2:**
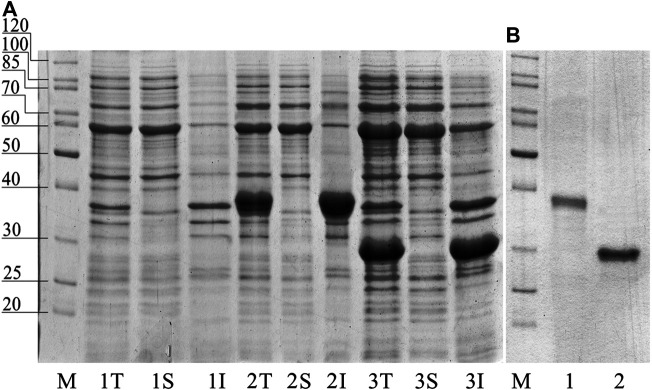
**(A)** SDS-PAGE of total (T), insoluble (I) and soluble (S) fraction of *E. coli Arctic express (DE3).* (1) non-induced control, (2) *E. coli Arctic express (DE3)* expressing Chit19; (3) *E. coli Arctic express (DE3)* expressing Chit19ΔChBD (3). **(B)** Refolded and purified Chit 19 (1) and Chit19ΔChBD (2).

pET plasmid system with strong T7-promotor was used for the Chit19 expression. The complete coding sequence of Chit19 and Chit19ΔChBD flanked by *Nde*I/*EcoR*I restriction sites was cloned using a cDNA library (described in *Isolation and cloning of the сhit19 gene* «Isolation and cloning of the *сhit19* gene») using primers Chit_NdeI, ChitΔChBD _NdeI and Chit_EcoRI (Supplementary Table S1). Thus, the genes encoding the Chit19 and Chit19ΔChBD region were cloned into the pET-28a vector using the *Nde*I/*EcoR*I restriction sites. *E. coli Arctic express (DE3)* and *E. coli Rosetta Gammi 2 (DE3)* producing strains were used. IPTG concentration and the temperature of induction were optimized to increase the expression level. The main pool of Chit19 accumulated in the insoluble fraction of the cell lysate, despite the recommendation to use both strains for the expression of cysteine-rich proteins. The yield of inclusion bodies of full-length Chit19 was 23.4 and 13.5 mg per 1 g of wet biomass in case of *E. coli Arctic express (DE3)* and *E. coli BL21(DE3) Rosetta-gammi 2*, respectively. Removal of the chitin-binding domain did not lead to an increase in protein solubility, apparently. Chitinases with and without a ChBD domain were expressed exclusively in insoluble form (Figure 2)**.**

The selection of strain allowed to achieve a two-fold increase the yield of the recombinant protein in the form of inclusion bodies. *E. coli Shaffle (DE3)* and *E. coli Arctic express (DE3)* surpasses the *E. coli BL21-CodonPlus (DE3)* strain in terms of the expression level of recombinant chitinase from *Drosera rotundifolia*. It was not possible to obtain a soluble form of the enzyme despite the use of thioredoxin labels and optimization of the temperature profile (Rajninec et al., 2020). Small amounts of the soluble form of the protein are possible during lysis of cells using buffers containing strong detergents (SDS) as it was shown in a latest work (Rajninec et al., 2020). The combination of this approaches enables to obtain active chitinase from *D. rotundifolia* with yields of about 1 mg of the target chitinase per Gram of wet biomass.

### Refolding Optimization

Using a variety of techniques to remove chaotropic agents is the key to a successful refolding. Refolding optimization was carried out in several steps. The effectiveness of various methods of removing the denaturing agent was evaluated at the first step. Three methods were tested: stepwise dialysis, rapid dilution, and stepwise refolding on Ni-Sepharose. We did not use any stabilizing additives in the refolding buffer at this stage.

Despite the fact that the dialysis method for refolding a plant chitinase was successfully used by Kirubakaran and Sakthivel, (2007), this technique was ineffective for gaining a high yield of refolded protein in our case. The method was time-consuming and leads to only up to 5% of the protein yield (the yield was estimated as the ratio of the mass of denatured protein before refolding to the mass of denatured protein obtained in soluble form after refolding); the main aggregation occurs when the concentration of urea decreases below 2M. The refolding process was quite simple when using Ni-Sepharose, and the protein yield was around 10%. The main protein aggregation occurs when it is washed out from the carrier. Presumably, this is due to the high local protein concentration at the front of the eluent, which leads to increased interaction between the protein molecules and its further aggregation. The method of rapid dilution showed the highest efficiency, with the 14.5% protein yield. Another important advantage of this method is the possibility of automation and scaling, which allows for multi-faceted optimization of refolding processes. However, it should be noted that solubility is not a criterion for the complete restoration of the biochemical properties of an enzyme, it turned out that the enzyme activity can differ by more than 25% (Table 1).

**TABLE 1:** Yield and specific activity of the refolded full-length chitinase from *D. capensis* (Chit19) obtained using various refolding methods.

Refolding method	Protein yield, %	Activity^a^, U/g
Dialysis	5.0 ± 1.1	200.3 ± 3.8
Rapid dilution	14.5 ± 1.6	323.8 ± 9.4
Ni-modified Sepharose	10.5 ± 1,4	184.4 ± 5.4

a The activity was determined towards colloidal chitin as a substrate. The hydrolysis reaction was carried out in 50 mM Na-acetate buffer (pH 5.0) for 30 min at 55°C.

### Effect of Chemical Additives on Refolding Efficiency

Excessive production of heterologous proteins in *E. coli* often leads to their improper folding and/or aggregation with the formation of inclusion bodies. This problem can be solved by optimizing the cultivation and induction conditions; however, it is almost impossible to find suitable conditions for hydrophobic proteins with multiple disulfide bonds. Typically, different approaches for converting inclusion bodies into a biologically active protein rarely provide sufficient refolded protein yield without proper optimization. The second stage of optimization was aimed to evaluate the effect of chemical additives on the refolding efficiency. Various chemical additives to the standard refolding buffer allow for a complex effect on the refolding process. Redox pairs of substances, such as glutathione, cysteine/cystine, accelerate the “shuffling” of disulfide bonds and reduce the refolding time (Hudson et al., 2015). Glycerol acts as a stabilizer of protein molecules by increasing the order of solvent molecules around the proteins. Increasing the concentration of glycerol improves the stability of the enzyme even at high protein concentrations. Although stabilizers such as glycerol or polyethylene glycol increase yield during refolding, protein aggregation can occur concurrently. Therefore, these types of supplements have always been used in combination with an aggregation inhibitor such as arginine (Yamaguchi and Miyazaki, 2014) or proline (Alibolandi and Mirzahoseini, 2011).

We screened the main additives in the basic refolding buffer (50 mM Tris-HCl, pH 8.5 and 200 mM NaCl), which proved to be effective in suppressing protein aggregation. Refolding buffers supplemented with 5% sucrose, 5% glycerol, 1% PEG 3000, 0.1M arginine, 1M proline, 0.5% Triton X100, 0.5% Tween 20 and 0.5% N-lauryl sarcosine sodium salt were tested (Supplementary Table S2). Testing was performed by refolding the full-length chitinase using the “rapid dilution” method in the buffer (see above). Tween-20 and Triton X100 did not significantly affect the yield of refolded chitinase, while the ionic detergent, N-lauryl sarcosine, decreased the yield of the recombinant protein compared to the control. Sucrose and PEG 3000 did not affect the refolding efficiency, while glycerol increased the chitinase yield. It should be noted that glycerol is the major buffer additive that has proven to be effective with a wide range of proteins and refolding techniques (Moghadam et al., 2015).

Proline and arginine can act as « chemical chaperones » by interacting with hydrophobic regions of the molecule (Meng et al., 2001; Xia et al., 2007). In our experiments, arginine showed a significant increase in the yield of refolded chitinase.

**FIGURE 3:**
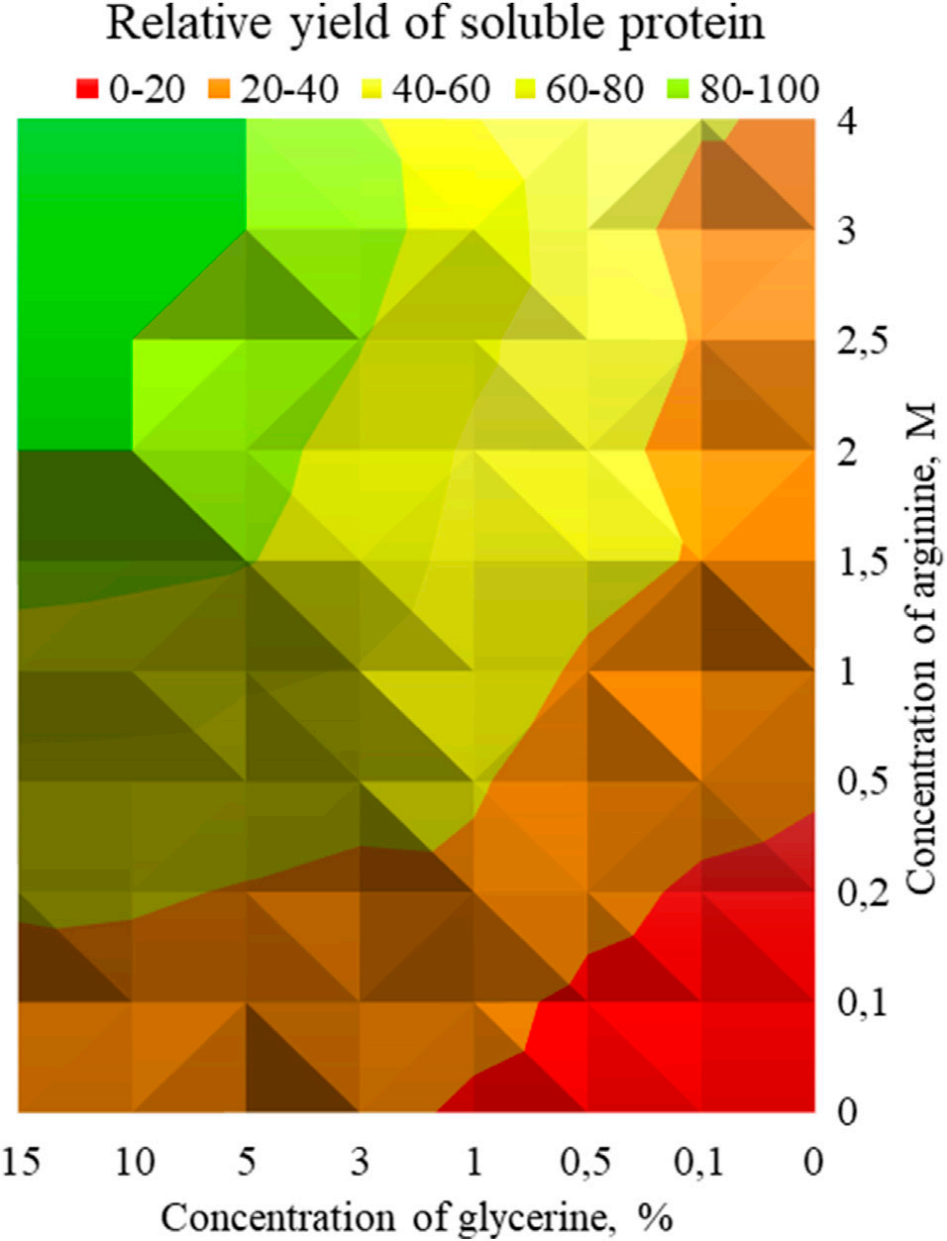
**-** Dependence of chitinase activity on the concentration of arginine and glycerol. Green zone—concentrations of glycerol and arginine, at which the maximum relative yield of active soluble protein was reached after refolding (80–100%), yellow-green zone (60–80%), yellow zone (40–60%), orange zone (20–40%) and red zone (0–20%).

Thus, two supplements were selected: glycerol and arginine. The effect of various concentrations of these additives on the effectiveness of refolding was investigated. Aliquots of chitinase dissolved in the denaturation buffer (100 μl) were added to 1 ml of refolding buffer (50 mM Tris-HCl, pH 8.0, 200 mM NaCl) supplemented with various concentrations of glycerol (from 0 to 15%) and arginine (from 0 up to 4 M). The enzyme activity was measured towards colloidal chitin in each sample. The diagram demonstrating the dependence of the chitinolytic activity on the glycerol and arginine concentration was created based on the data obtained. (Figure 3).

It should be noted that glycerol and arginine displayed a synergistic effect, the maximum activity of 360 ± 4.6 U/g was achieved at 10% glycerol and 2M arginine with the 25% yield of refolded protein. Further increase in their concentration did not affect the efficiency of refolding.

The next stage of optimization was the selection of redox agents that accelerate the formation of proper disulfide bonds. The following additives were tested in this work: 1) 2-mercaptoethanol (1–5 mM), 2) oxidized glutathione/reduced glutathione (3 mM/0.3 and 1/1 mM), 3) cysteine/cystine (0.1/3 and 0.5/5 mM). The addition of mercaptoethanol and redox pair of cysteine/cystine in the used concentration range did not lead to a significant change in the yield of the soluble form of the protein and activity. Of all the above, only glutathione at a concentration of 0.3/3 mM had a positive effect on the yield of soluble chitinase, which increased up to 30%. Previously, the effectiveness of using redox pairs GSH/GSSG has been shown elsewhere (Wang et al., 2006; Bastings et al., 2008). However, most often this system influenced not so much the yield of soluble protein, but rather its activity, directing the formation of disulfide bonds in the “right” direction. Thus, the selected optimal composition of the refolding buffer was 50 mM Tris-HCl (pH 8.0), 100 mM NaCl, 10% glycerol, 2 M arginine, 3 mM oxidized glutathione and 0.3 mM reduced glutathione. The yield of the soluble full-length chitinase was 30%. The protocol was also implemented for Chit19ΔChBD, where the yield of soluble protein was 42%.

### Analysis of Modeled 3D-Structure of Chit19 and *in Silico* Design of Variants

**FIGURE 4:**
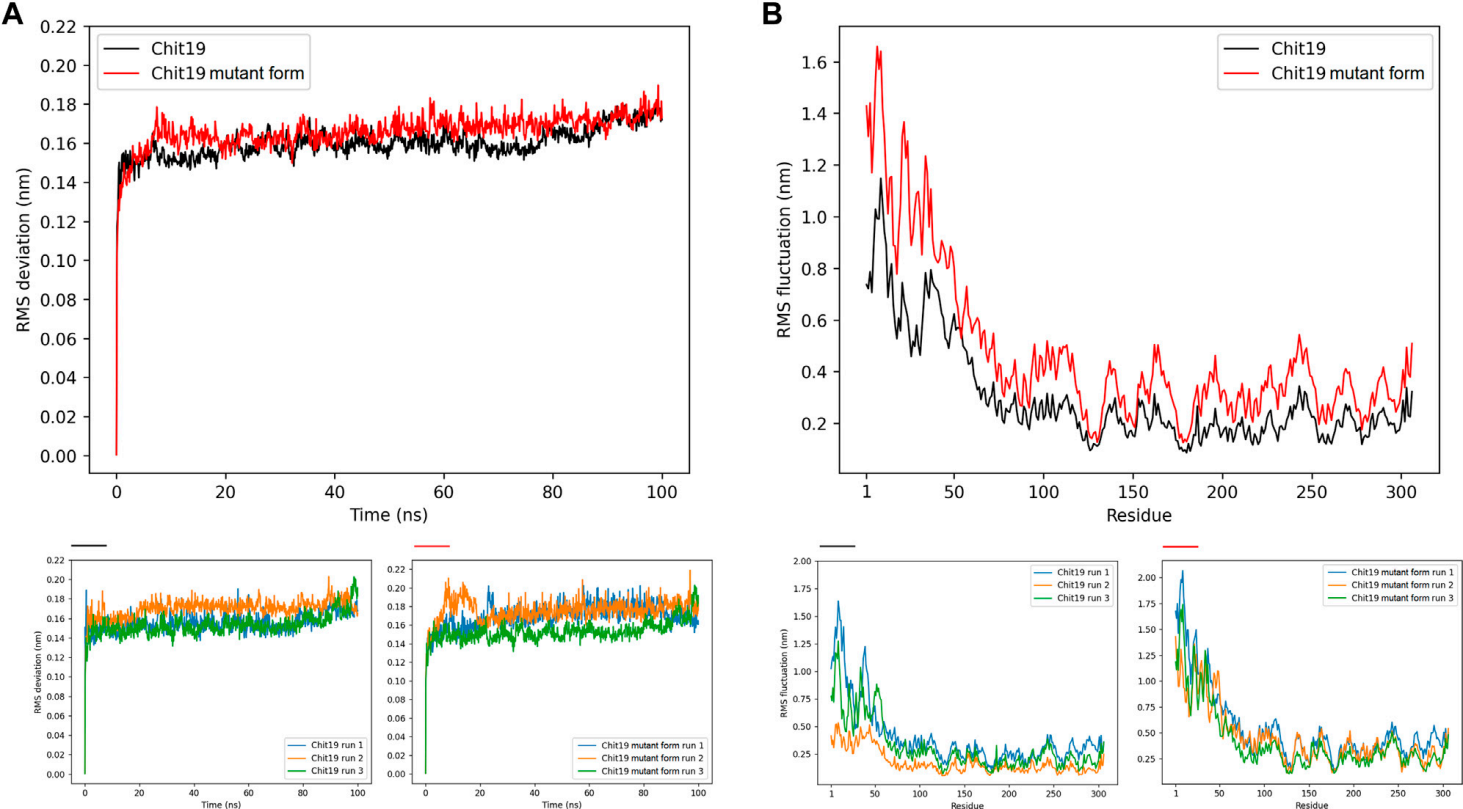
**(A)** Backbone RMSD averaged for three runs for positions 62–306 (excluding ChBD-linker region as the linker region had high fluctuations throughout the simulation runs) of Chit19 wild-type (black) and variant C191A/C231S/C286T (red) over simulation time in ns. **(B)** RMSF per residue averaged for three runs of all 306 residues of Chit19 wild-type (black) and variant Chit19- C191A/C231S/C286T (red). Residue pos. 1–39 define the ChBD and pos. 59–84 the Chit19 linker region. Smaller diagrams below indicate RMSD **(left diagrams)** and RMSF **(right diagrams)** values for Chit19 wild-type and variant for each single simulation run.

The Chit19 homology model was analyzed using the software VERIFY3D (Bowie et al., 1991; Lüthy et al., 1992) and PROCHECK (Laskowski et al., 1993) and passed the quality assessment based on internal scoring functions (Supplementary Figure S2). By aligning the model structure with PDB structure 2DKV, we identified the positions Glu 130 and Glu 152 as putative catalytic residues of the Chit19 model based on the studies of Kezuka et al. (2010) and Huet et al. (2008). Further, evolutionary conservation of amino acids analysis revealed a high conservation of the catalytic residues and the helices and loops forming the catalytic core (for residual conservation details of Chit19 sequence see Supplementary Figure S5). Similar insights were also obtained capturing the evolutionary trace of Chit19 (Supplementary Figure S6). MD simulations confirmed the overall remained stability of the predicted model (based on root mean square deviations (RMSDs) calculated for the core domain of the model, Figure 4)

**FIGURE 5:**
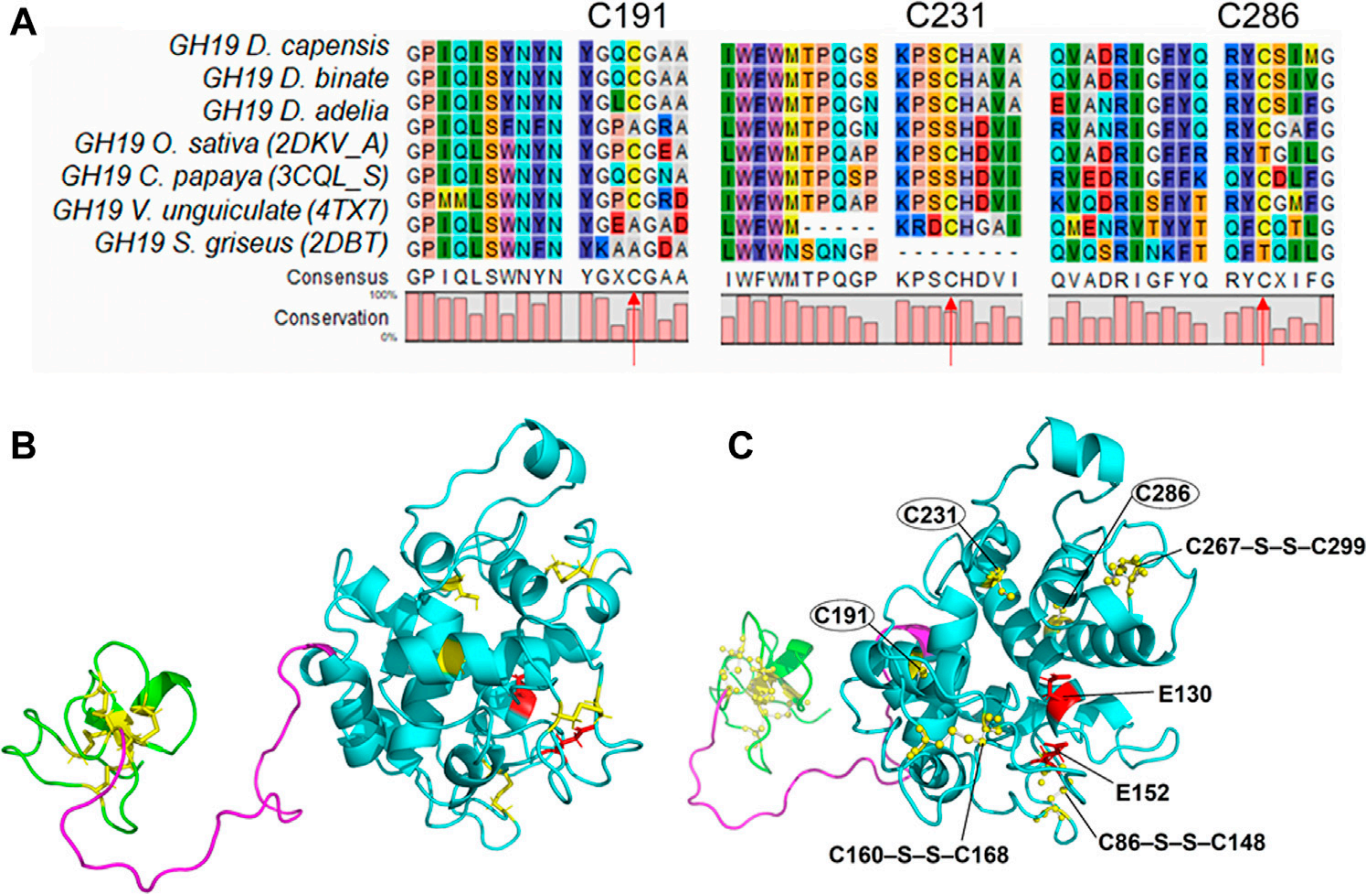
Protein alignment of homologous chitinases in the areas of unbonded cysteine**s:** **(A)** Alignment of the seven chitinase sequences that were selected. **(B)** Structure of Chit19 homology model with the three domains including chitin binding domains (ChBD, residues 1–40, green), linker (residues 41–65, magenta), and core domain (residues 68–306, cyan), and highlighted disulfide bonds (yellow) and putative catalytic residues (E130 and E152, red)). Secondary structures are highlighted as ribbon models (indicating α-helices, ß-sheets, and loop regions). **(B)** and **(C)** represent the same structure but in **(C)** the snapshot angle is rotated to highlight the putative catalytic sites and the disulfide bridges (balls and sticks) of the core domain (C86–S–S–C148, C160–S–S–C168, C267–S–S–C299) as well as the free cysteines (C191, C231, C286).

To determine possible substitutions for the selected cysteines, we performed multiple alignment of chitinases of the 19th family available in the PDB database (*Oryza sativa* 2DKV*, Carica papaya* 3CQL*, Vigna unguiculata* 4TX7*, Brassica juncea* 2Z37*, Bryum coronatum* 3WH1*, Streptomyces griseus 2DBT*) (Figure 5). Conservation among these seven sequences (as well as the alignment to 100 non-redundant sequences; Supplementary Figure S5) showed high conservation of the cysteines of the catalytic core domain, i.e., model positions C191, C231, C286, while also Ala, Ser, respectively. Thr showed minor conservation at these positions (Figure 5). Structural conformations of proteins can drastically alter properties as function, solubility, and aggregation–among many more–that, in this regard, can be associated to the thermodynamic stability ($\text{Δ}G_{\text{unfolding}}$) of the variant (Lee and Blaber, 2009). It has been described that substitutions of Cys effectively changed protein structures to maintain or include novel H-bond interactions–resulting in a local shrink/collapse, e.g., for substitutions to isosteric Ser or polar Thr accommodating for the smaller oxygen radius compared to the radius of Cys Sγ, or in a structural expansion, e.g., substitutions to nonpolar amino acids like Ala, respectively (Xia et al., 2015). Amino acid substitutions to Thr and Ser were experimentally tested at the identified positions to enhance Chit19 solubility, as these substitutions are conserved among the aligned sequences and/or are polar as well as sterically similar to Cys.

### Site-Directed Mutagenesis and Protein Expression

**FIGURE 6:**
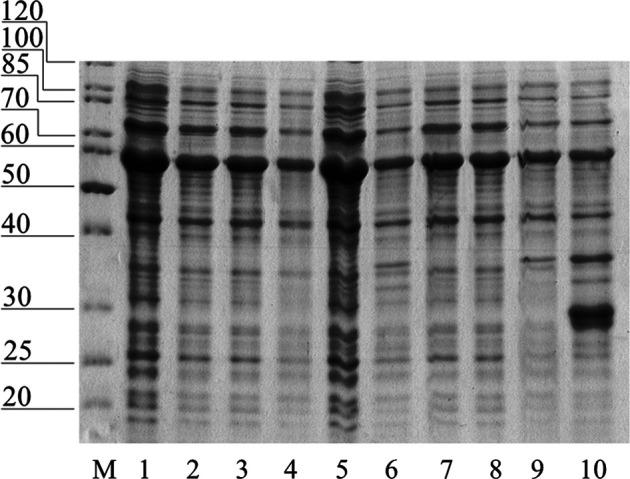
Soluble fraction of *E. coli* after expression of mutant forms of Chit19: **1—**Chit19, **2—**Chit19(C191A), **3—**Chit19(C231S), **4—**Chit19 (C286T), **5—**Chit19(C191A, C231S, C286T), **6—**Chit19ΔChBD, **7—**Chit19ΔChBD (C191A), **8—**Chit19ΔChBD (C231S), **9—**Chit19(C286T), **10—**ChitΔChBD (C191A, C231S, C286T)

Removal of free cysteines will reduce the number of conformations and disulfide bonds that proteins can form. Also, this reduces the level of intermolecular interactions in the case of surface cysteines. Removal of the three free cysteines from the catalytic core and the cysteine-rich chitin-binding domain allowed the successful expression of GH 19 chitinase in *E.coli* (Figure 6). It was a complete protein with catalytic activity, despite the fact that the yield did not exceed 50 mg/L. The mutation of three cysteines did not lead to a change in the level of activity compared to the non-mutant protein and amounted to 121.4 U/g, determined by colloidal chitin.

The first step was *E. coli* strains selection and expression conditions optimization. The second step is to develop an optimal refolding technique in the case of protein expression in the form of inclusion bodies. One of the bottlenecks for the *E. coli* expression system is the absence of a processing system for the formation of disulfide bonds. (Berkmen, 2012). Free cysteines often lead to the formation of aberrant disulfide bonds in the case of protein expression in *E. coli*. One of the most effective solutions to obtain a higher yield of properly oxidized soluble protein is the mutation of nonessential cysteines, which form aberrant disulfide bonds (Zhang et al., 2010). The second solution to this problem is the expression of the protein in a mutant *E. coli* strain with alternative mechanisms of oxidative folding. The process of protein folding in *E. coli* is significantly influenced by the cultivation conditions: temperature, the presence of growth factors and chemical agents in the growth medium (Alibolandi and Mirzahoseini, 2011).

### Molecular Basis of Wild-type and Improved Variant

MD simulations of wild-type and improved variant (triple mutant) revealed higher RMSDs computed for the Chit19 model core domain (residues 62–306) and increased RMS fluctuations (RMSF) computed across the full sequence for 100 ns simulation time and three runs (Figure 4). However, both structural models remained overall stability after system equilibration (RMSD values in the range of 0.16–0.18 nm; Figure 4A). MD trajectory analyzes revealed that the core domain of Chit19 remained stable throughout the runs, while the substituted variant’s core domain was more flexible in total (Figure 4A). Further, computations revealed that the ChBD domain was highly fluctuant yielding RMSF values of about 0.5–1.15 for wild-type variant and increased RMSF values of about 0.8–1.65 nm for the triple substituted variant, while also for the linking region a similar trend was observed yielding RMSF values of about 0.3 and 0.4 nm for wild-type and the substituted variant, respectively (see fluctuations of residues 59–86, Figure 4B). A higher fluctuation of individual residues of the substituted variant during the simulations across all 306 residues (Figure 4B) is evident.

MD simulations showed that the substituted variant loses structural stability and gains flexibility relative to the wild-type enzyme. Such enhanced fluctuation behavior of the substituted variant is likely caused by the substitution of the free cysteines of the substituted variant, while the fluctuation is generally highly increased for the linker and binding domain because the removal of free cysteines will reduce the number of conformations and (interdomain) bonds that proteins can form. Also, in the case of surface cysteines, this could reduce the level of intermolecular interactions, while we observed, that the residual solvent accessibility of the cysteines was very low and potentially only C286 contributes to the Chit19 solvent accessible surface. Further, inter-residual chain interactions of free cysteine sulfur atoms were reported to contribute to the tertiary structure stability, e.g., *via* inter-residue S∙∙∙C=O and S∙∙∙N interactions (Pal and Chakrabarti, 1998). Thus, substitutions of these free cysteines might induce lower protein stability (Zheng et al., 2017) and could be an indication of the higher flexibility observed for the substitution variant in contrast to the wild type. Besides, the intra- or inter-residual interaction of free cysteines could also limit the solubility of Chit19 by causing any undesired contacts as observed for C-terminal cysteines (Schmiedl et al., 2000).

### Evaluation of the Functional Characteristics of the Chit19

The characteristic feature of GH 19 chitinases is the dualism of properties. Fungicidal activity is an important characteristic in addition to their catalytic properties. The specific activities were determined for colloidal and crystalline chitin, chitosan (140 kDa) for the obtained homogeneous Chit19, Chit19ΔChBD and three-point mutant Chit19ΔChBD (Table 2). Full-sized Chit19 has a specific activity of 360 ± 4.6 U/g for colloidal chitin, and 84,5 ± 3.9 U/g for crystalline chitin **(** Table 2), which is comparable to the activity of chitinase from *Dionaea muscipula* (Paszota et al., 2014). However, the activity of the obtained chitinase is significantly lower compared to the chitinase of *Drosera rotondofula*, which may be associated with the peculiarities of the used measurement methods and the properties of chitinase itself. Despite significantly higher catalytic activity, chitinase from *D. rotondofula* has the weakest thermal stability, a low temperature optimum, and a narrow pH-optimum (Rajninec et al., 2020). In our previous work, we demonstrated that the optimum action of the chitinase from *Drosera capensis* is 55°C and pH 5.0 (Sinelnikov et al., 2020).

**TABLE 2:** specific activities of the native and mutant forms of Chit19.

Enzyme	Substrate	Specific activity^a^, U/g
Chitinase from *Drosera capensis* (Chit19)	Colloidal chitin	360.0 ± 4.6
	Crystalline chitin	84.5 ± 3.9
	Chitosan 140 kDa	65.4 ± 2.4
Chitinase from *Drosera capensis* without ChBD domain (Chit19ΔChBD)	Colloidal chitin	130.0 ± 3.2
	Crystalline chitin	**—**
	Chitosan 140 kDa	67.0 ± 5.4
Chitinase from *Drosera capensis* without ChBD domain and three-point mutant (ChitΔChBD (C191A, C231S, C286T)	Colloidal chitin	121.4 ± 8.5
	Crystalline chitin	**—**
	Chitosan 140 kDa	60.3 ± 1.4

a The hydrolysis reaction was carried out in 50 mM Na-acetate buffer (pH 5.0) for 30 min at 55°C.

Removal of the chitin-binding domain resulted in a more than 3-fold decrease in catalytic activity. It should be noted that in a similar study of chitinase GH19 from tobacco (Iseli et al., 1993), the removal of the chitin-binding domain did not lead to a change in catalytic activity, but significantly reduced the fungicidal activity. Such a contradiction is not uncommon in studies of the effect of a chitin-binding domain on activity. The contribution of the chitin-binding domain to the catalytic activity of family 19 chitinases may differ and requires additional research in each individual case.

**FIGURE 7:**
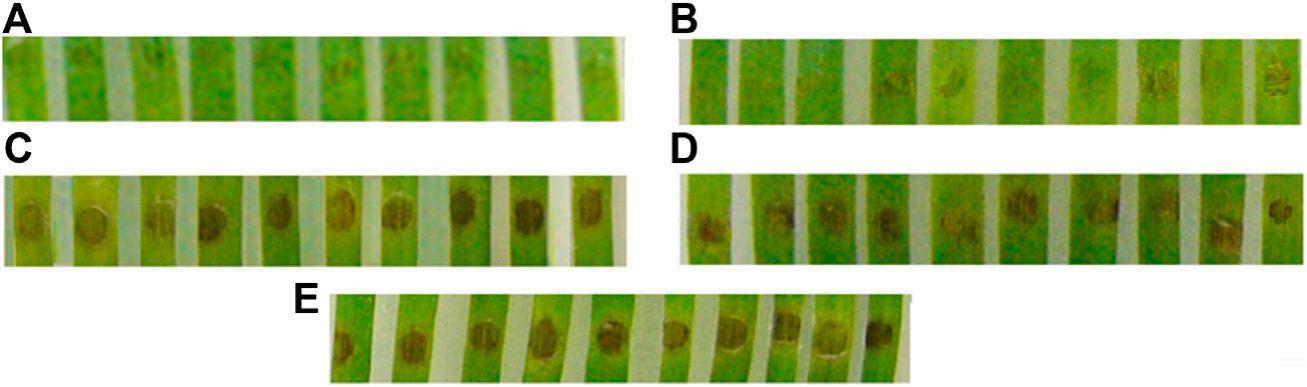
**–** Mitigation of disease symptoms on leaf sections cut from detached wheat leaves due to refolded enzyme application. Upper (distal) pats of leaf sections inoculated with a suspension of *Parastagonospora nodorum* conidia containing: **(A)** Chit19, **(B)** Chit19ΔChBD; **(C)**, **(D)**—lower (basal) parts of the same leaf sections inoculated with conidia suspended in Na-acetate buffer; **(E)** basal parts of control leaf sections inoculated with *P. nodorum* conidia suspended in distilled water. Representative photographs of one of three experiments.

The fungicidal activity of Chit19 is a consequence of their high affinity for the cell wall of the phytopathogen. Therefore, restoring the original structure is an important condition for proper functioning. The use of these refolding approaches makes it possible to obtain fully functional chitinase both in terms of enzymatic activity and fungicidal activity. Chitinase after refolding was fully functional and effectively prevented disease development on detached wheat leaves. Inoculation of leaf sections with *P. nodorum* conidia pre-exposed to both Chit19 and Chit19ΔChBD resulted in significant mitigation of the disease symptoms compared to controls **(** Figure 7).

## Conclusion

The main goal of *in vitro* refolding of a recombinant protein from bacterial inclusion bodies is to obtain a soluble form of the target protein; however, there is no single refolding method that gives satisfactory refolding results for different proteins. In this work, we have chosen the optimal composition of the refolding buffer and the optimal concentrations of arginine and glycerol to obtain the maximum yield of soluble Сhit19. The effect of redox pairs on the yield of soluble protein was evaluated. Such a strategy, most likely, can be successfully applied to refolding other « problematic » recombinant proteins.

Thus, the composition and optimization of the concentration of supplements is an important step towards successful protein refolding and must be done on a case-by-case basis. This work shows how optimization helped to increase the yield of the soluble form of chitinases by more than three folds compared to the basic technology. A soluble form of the enzyme was obtained in *E. coli* after replacing three free cysteines in the core domain for the Chit19ΔChBD. These results indicate that replacement of free cysteines maybe is a good strategy for solving problems of insoluble expression in *E. coli*. The catalytic properties of the obtained chitinase are lower compared to the closest chitinase homologue from *D. rotondofula*. However, *D. capensis* chitinase is much more stable at elevated temperatures and at a wide pH range, while maintaining its phytoprotective properties. A more detailed analysis of the differences between these chitinases will lead to development of advanced forms of the enzyme with high catalytic activity and better stability. In the future, the soluble chitinase can be used as a separate biofungicide or enhance the effect of existing biofungicides available on the market, such as Bt-σ-endotoxins, to control plant pathogenic fungi or insects, and nematodes. However, despite obtaining a soluble form of mutant chitinase, obtaining large amounts of native chitinase for agricultural needs remains a challenges that can most likely be tackled using eukaryotic expression systems.

## Data Availability

The datasets presented in this study can be found in online repositories. The names of the repository/repositories and accession number(s) can be found in the article/Supplementary Material.
