# Supplementary material for: Expression and Refolding of the Plant Chitinase From Drosera capensis for Applications as a Sustainable and Integrated Pest Management
Source: Front Bioeng Biotechnol. 2021 Sep 21;9:728501. doi: 10.3389/fbioe.2021.728501 (PMC8490864; doi:10.3389/fbioe.2021.728501)
Supplement: Supplementary file 1 [file Table1.docx]

**SUPPLEMENTARY MATERIALS**

| **Section** | **Page** |
| --- | --- |
| Chitinase cloning | S1 |
| Refolding of chitinase | S2 |
| Homology modeling | S2 |
| Evolutionary conservation analysis | S5 |
| Evolutionary trace studies | S7 |
| Molecular dynamics simulations | S8 |

**Chitinase cloning**


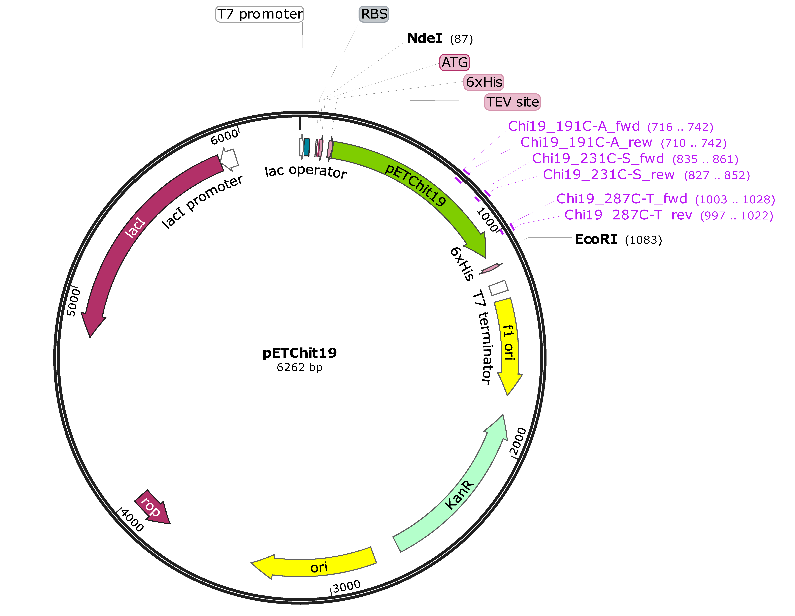

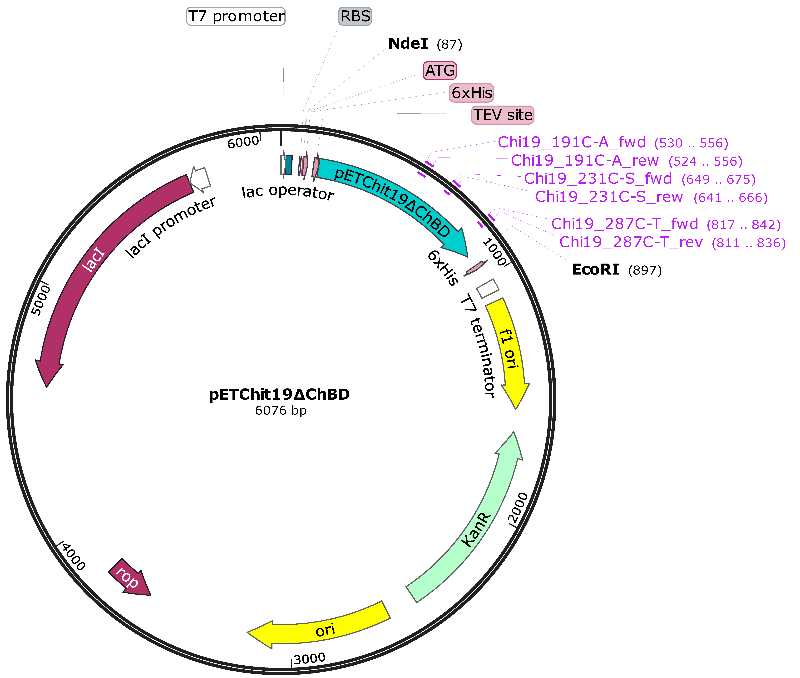


**B**

**A**

**Figure S1 – The map of plasmids: pETChit19 (A) and pETChit19ΔChBD (B)**

Amplification conditions: primary denaturation 98^o^C-60s, denaturation in a cycle 98^o^C - 8 s, annealing of primers 50^o^C - 30 s, elongation 72^o^C - 120 s, 15 cycles

**Table S1 – Primers for *chit19* cloning and site-directed mutagenesis**

| **Primer name** | **Sequence 5’-3’** |
| --- | --- |
| ChitFS | acygcaccmgatggtccatatgcwtg |
| ChitRS | gcrtcatccaaaaccatatmgytgtt |
| Chi19_С287T_rev | ccataatgctggtgtatctctggtag |
| Chi19_С287T_fwd | gagatacaccagcattatgggaatcg |
| Chi19_С231S_rew | gcatggcttgagggcttgctcccttg |
| Chi19_С231S_fwd | caagccctcaagccatgctgtcgccac |
| Chi19_С191A_rew | gcagcgccggcttgcccatagttgtag |
| Chi19_С191A_fwd | tatgggcaagccggcgctgccatcaac |

***Y -** C or T; **W** - A or T; **M -** A or C; **R -** A or G

**Refolding of chitinase**

**Table S2 - Evaluation of the chemical additives effect in refolding buffer on refolding efficiency.**

| **Impact on the effectiveness of refolding** | | | | | | | | |
| --- | --- | --- | --- | --- | --- | --- | --- | --- |
| sucrose | glycerol | arginine | proline | PEG 3000 | Triton 100x | Twin-20 | N-Lauryl sarcosine |  |
| n | + | + | n | n | n | n | - |  |

“+” - increase in refolding efficiency,

“-” - decrease in refolding efficiency,

“n” - no effect

**Homology modeling**

The homology model of Chit19 sequence (residues 20 – 325, 306 residues sequence length) was constructed using the homology modeling macro(E. Krieger, Nabuurs, & Vriend, 2003) of the YASARA software suite (v. 19.12.14.W.64) with default parameters.(Abraham et al., 2015; Elmar Krieger, Koraimann, & Vriend, 2002; Elmar Krieger & Vriend, 2014; Land & Humble, 2018) Signal peptide analysis using the Signal-P 5.0 webserver revealed that a signal peptide cleavage site is between pos. 20 and 21 (TYA-VQ) with a probability of 96.31 %.(Almagro Armenteros et al., 2019) In the following we refer to the model sequence numbering starting at position 20 of the full Chit19 sequence (see **Figure S2**). Possible templates for modeling were identified by running 5 PSI-BLAST iterations to extract a position specific scoring matrix (PSSM) from UniRef90, and then searching the protein data bank (PDB) for a match (i.e., hits with an E-value below the homology modeling cutoff of 0.1).

The best homology model of Chit19 (Z score = 0.078) was generated solely based on the template structure of a chitinase from *Oryza sativa* (PDB identifier 2DKV-A, hereinafter only referred to as 2DKV), resolution 2.0 Å;(Kezuka et al., 2010) sequence identity of 67% to Chit19, aligned to Chit19 residues 21-325 with 96% coverage using blastp’s BLAST 2 SEQUENCES.(Johnson et al., 2008; Tatusova & Madden, 1999) The numbering of the model and the full Chit19 sequence can be perceived from the YASARA alignment taken for modeling, see **Figure S2**. Further, the loop connecting residues 39 to 69 (residues QCGGS–SPPPAPPSPTPSPPSPSGGG–DVSSI) was modeled by YASARA using a non-redundant subset of the PDB with 90% sequence identity cutoff.(Wang & Dunbrack, 2003) The resulting homology model of Chit19 is shown in **Figure S2**; the first residue of this model is pos. 20 (N-terminus-AVQC…). Based on the structural superposition (structural alignment in YASARA) to 2DKV and on the analyses of Kezuka *et al.*(Kezuka et al., 2010) and of Huet *et al.,*(Huet et al., 2008), the putatively identified catalytic amino acids – glutamic acid (Glu) residues acting as an acid and a base catalyst – are located at the bottom and the side face of the catalytic cleft – here at positions Glu 130 and Glu 152.

**
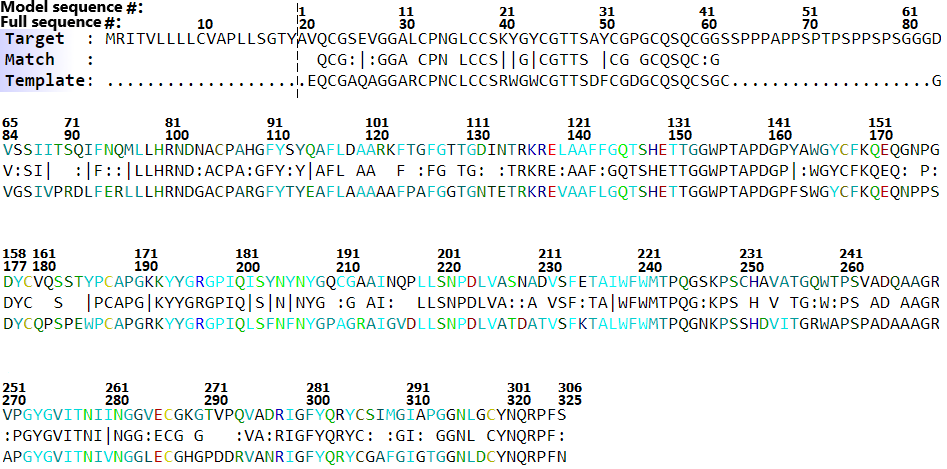
**

**Figure S2.** Aligned sequences of Chit19 (target) and sequence of structure 2DKV-A (template) and matching amino acids (match) taken from the YASARA homology modeling report. The homology model sequence covers the sequence after the dotted line (target model sequence pos. 1 – 306). The sequence numbering represents Chit19 full sequence and model sequence numbering.

The homology model was evaluated using the Structural Analysis and Verification Server (SAVES) that implements structure quality assessment via tools as VERIFY3D (Bowie, Lüthy, & Eisenberg, 1991; Lüthy, Bowie, & Eisenberg, 1992) and PROCHECK (Laskowski, Macarthur, Moss, & Thornton, 1993). VERIFY3D revealed that 100.00% of the residues have an averaged 3D-1D score of >= 0.2; the Ramachandran plot and main-chain parameter analyses of PROCHECK revealed that all except one structural model parameters fulfill the criteria of a good quality model (see **Figure S3**).


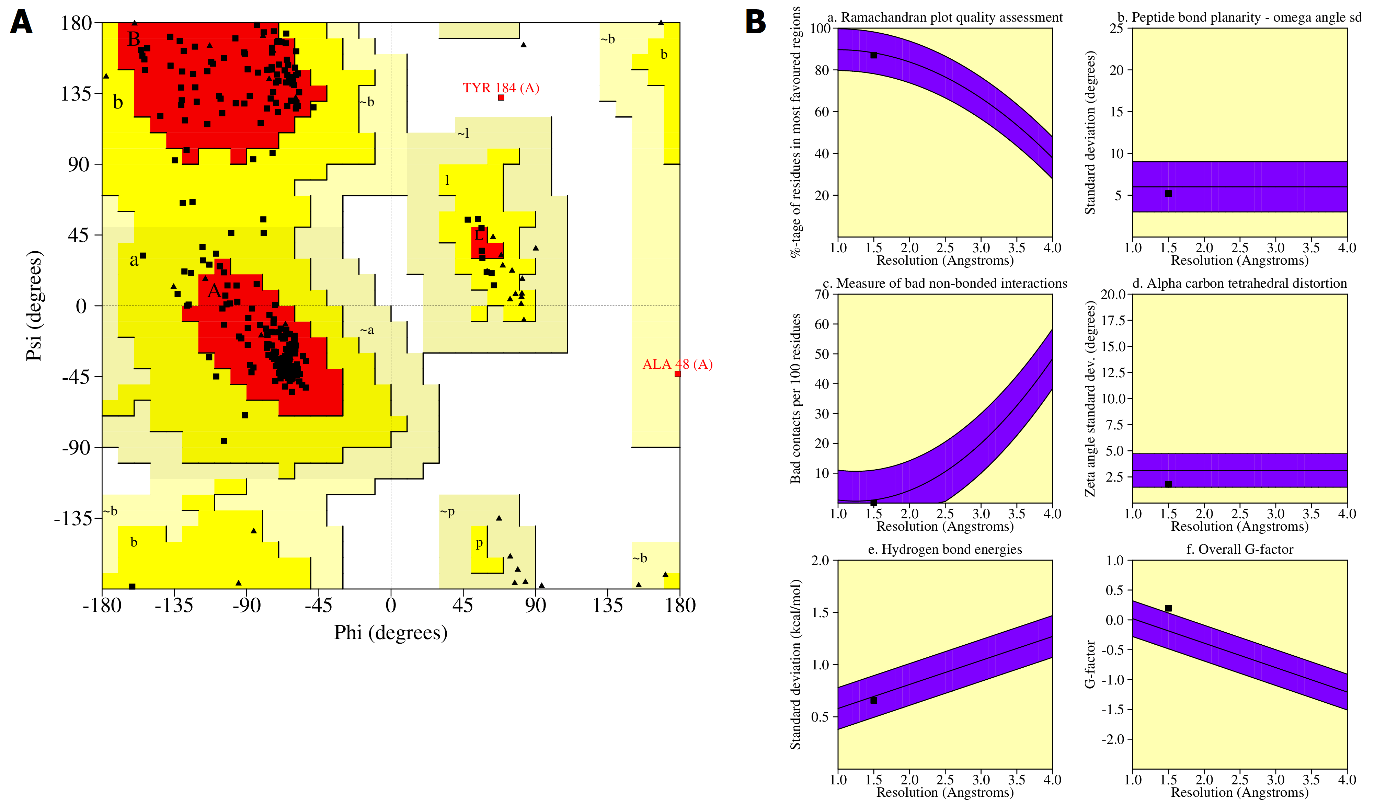


**Figure S3.** SAVES v.6 validation of the Chit19 homology model. **A**) Ramachandran plot of the Chit19 homology model denoting backbone dihedral angles φ and ψ in degrees: 87.1% of all residues are in favored regions, 12.5% in allowed regions, and 0.4% in disallowed regions. **B**) PROCHECK main-chain parameters. Graphs represent (a) quality assessment of Ramachandran plot (87.1%, inside accepted purple region), (b) peptide bond planarity (5.2 degrees, inside accepted region), (c) measure of bad non-bonded interactions (0/100 residues), (d) alpha carbon tetrahedral distortion (1.8 degrees, inside accepted region), (e) hydrogen bond energies (0.7 kcal/mol, inside accepted region) and (f) overall G-factor (0.2, outside accepted region)].

# **Evolutionary Conservation analysis**

Evolutionary conservation analysis of the Chit19 sequence was performed using the ConSurf webserver with standard parameters for multiple sequence alignment (MSA), phylogenetic tree building, and conservation scores. The result is presented in **Figure S4**, indicating that the ChBD (residues 1 – 39) is highly conserved as well as the core domain (residues 66 – 306). It should be noted that the linker domain (residues 40 - 65) is not highly conserved among the aligned sequences. The linker residues are rich in prolines (38.5%; 10/26 residues) followed by serines (26.9%; 7/26) and glycines (19.2%; 5/26), potentially causing a high conformational flexibility of the interdomain linker region providing a potentially higher variability of domain arrangement as observed by molecular dynamics (MD) simulation studies.


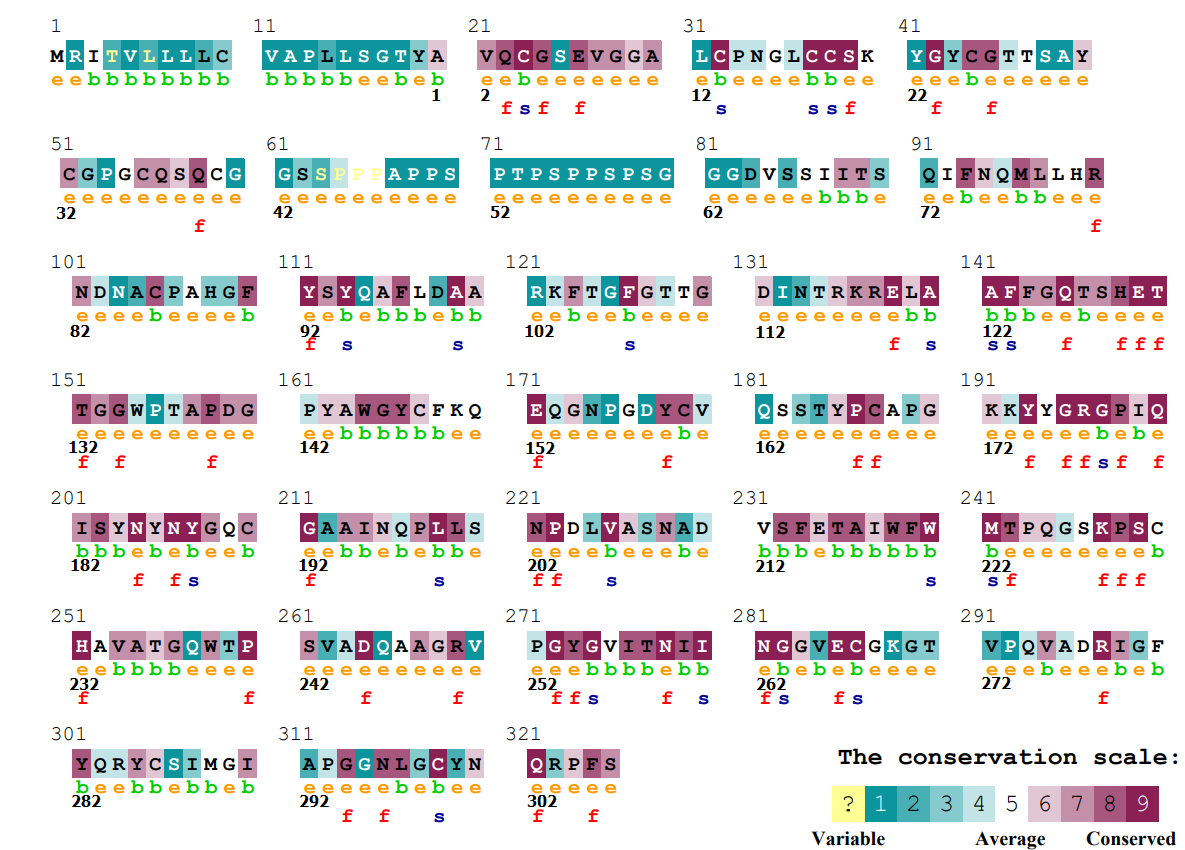


**Figure S4.** ConSurf-based evolutionary conservation analysis of Chit19 amino acids as computed using the default ConSurf MSA settings (protein databank: UniRef90, search algorithm: HMMER, MSA construction method: MAFFT, totally 150 sequences aligned), colored by the conservation scale as indicated (blue represents a variable residue conservation, white an average level of conservation, and red a highly conserved residue). Letters ‘e’ and ‘b’ represent predicted exposed and buried positions, respectively. Residues that are predicted to have either high structural or functional importance are denoted by ‘s’ and ‘f’, respectively. Sequence numbering on top represents the full Chit19 sequence and below (bold) the numbering of the homology model sequence.

To further elucidate in more detail the conservation and patterns of non-redundant aligned sequences for Chit19 we performed gapped sequence alignment of 100 non-redundant sequences using the Chit19 full sequence as input for protein database search using the BLAST webserver using default parameters. The gapped alignment was subsequently provided as input for the WebLogo 3 webserver to create a sequence conservation logo of aligned sequences. We perceived that all cysteine positions of the Chit19 core domain showed very high conservation among the aligned sequences (see **Figure S5**). In addition, there was less conservation of ChBD cysteine positions between sequences from the alignment. Free cysteines of the core domain (C191, C231, C286) were identified by visual inspection of the Chit19 model in YASARA. Although the three cysteines are in very close proximity to the protein surface, the visualization of the solvent accessible surface area in YASARA surface using the default parameter for modeling revealed that only C286 contributes marginally to the solvent accessible surface area of the protein.


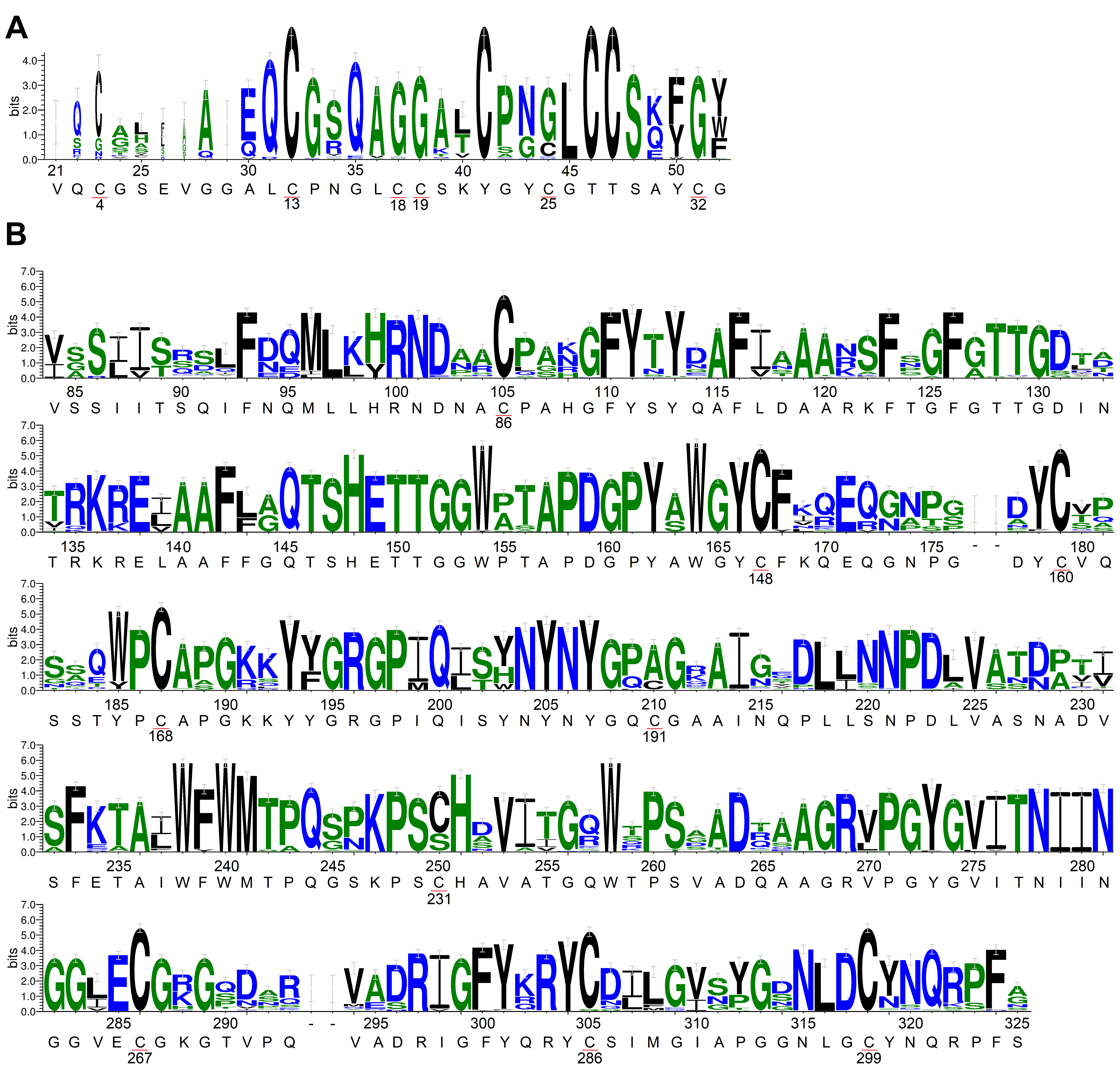


**Figure S5.** Conservation analysis of Chit19 full sequence (small black letters and sequence numbers) aligned to 99 non-redundant sequences of an gapped MSA using the BLAST webserver visualized in WebLogo3. Chit19 cysteines are highlighted with red lines and additionally labeled with the sequence number of the Chit19 homology model below. Sequence conservation is shown in bits, the size of each letter represents the conservation, i.e., frequency, of this amino acid at the corresponding position among the alignment of the 100 protein sequences. The color of the amino acid letters represents their hydrophobicity as defined by the WebLogo3 default options (hydrophilic: RKDENQ, blue; neutral: SGHTAP, green; hydrophobic: YVMCLFIW, black). (A) represents sequence conservation of residues of the ChBD region (here shown pos. 21 – 52) and (B) of the core domain (here shown pos. 84 – 325) of the sequence alignment.

**Evolutionary trace studies**

The evolutionary trace (ET) webserver (Wilkins, Erdin, Lua, & Lichtarge, 2012) was applied to predict evolutionary conserved and functional sites of the Chit19 full sequence. The multiple sequence alignment was performed using the standard parameters of the ET webserver resulting in 383 input sequences for the sequence alignment for Chit19. Evolutionary scores for residues are calculated with the reciprocal real-value evolutionary trace (rvET) method (Mihalek, Reš, & Lichtarge, 2004) and visualized in Figure S6. Similar to results obtained from ConSurf- and WebLogo-based studies, also ET studies revealed, based on the evolutionary context of Chit19, lower importance of the linking (and sequence peptide) region, i.e., lower conservation of amino acids in this aligned region based on simulated evolution-derived sequences. In addition, also the putative catalytically active glutamic acids (pos. 130 and 152 of the model sequence) are regarded as highly important throughout the evolved sequences, while also utmost residues of the core domain (starting from residues ~62 of the model sequence) and especially the active site cavity residues (pos. 121 – 141, 174 – 189, 214 – 222, 252 – 267 of the model sequence) are classified as highly important.


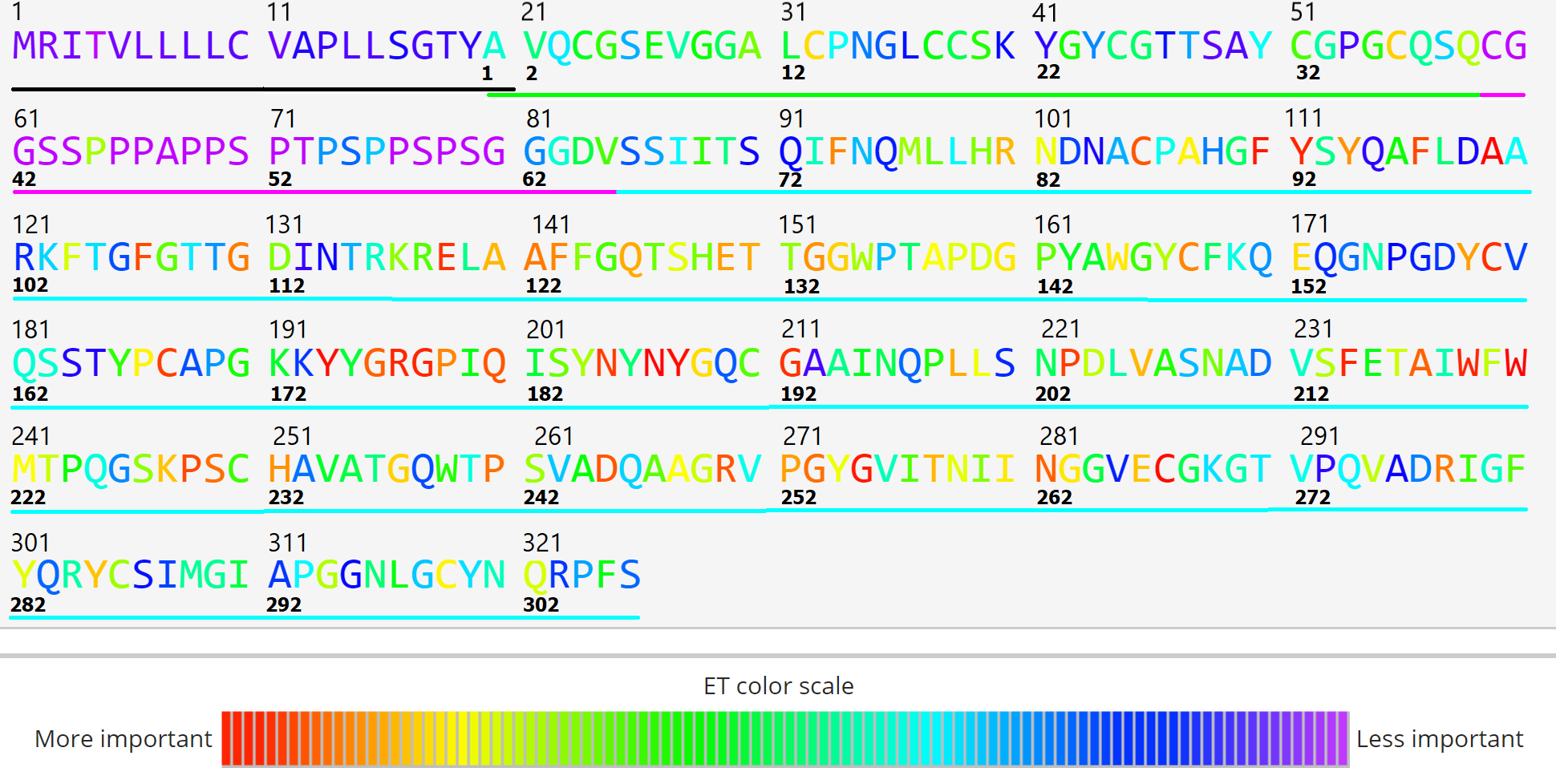


**Figure S6.** Full length Chit19 sequence colored by their relative importance based on the rvET method according to the coloring scheme following the level of importance corresponding to the scheme red>yellow>green>blue>purple. Numbering on top of the sequence represents the full Chit19 sequence numbering and below (bold) the numbering of the homology model sequence. Lines present protein and sequence domains and regions (signal peptide, black; ChBD, green; linker, magenta; core, cyan).

# ***In silico* mutagenesis and molecular dynamics (MD) simulations**

MD simulations were performed to compare the stability of the substituted variant with improved solubility (Chit19-C191A/C231S/C286T) with Chit19 wild-type. We analyzed the flexibility of both variants using molecular dynamics runs performed with the GROMACS 2019 software package (Abraham et al., 2015; Berendsen, Van Der Spoel, & Van Drunen, 1995; Van Der Spoel et al., 2005). The substituted variant was modeled using the FoldX method (Schymkowitz et al., 2005) available through the FoldX plugin (Van Durme et al., 2011) in YASARA. Side chain protonation states were set using the *pdb2gmx* tool implemented in GROMACS based on calculation of the p*K*_a_ values of the side chains at pH 7.0 using the PROPKA method(Li, Robertson, & Jensen, 2005) implemented in the PDB2PQR webserver.(Dolinsky et al., 2007) MD runs were performed each in isothermal-isobaric (NPT) ensemble after system charge neutralization, energy minimization, and canonical (NVT) equilibration ensemble using the SPC/E water model (Berendsen, Grigera, & Straatsma, 1987) and the AMBER99SB-ILDN force field.(Lindorff-Larsen et al., 2010)

Prior to simulations, an energy minimization was performed for energy-efficient positioning of water molecules using the steepest descent minimization algorithm reaching a minimum energy level of 1,000 kJ mol^-1^ nm^-1^ under position restraining of heavy protein atoms. After energy minimization and following system heating, the systems were equilibrated under NVT ensemble at 300 K by execution of the modified Berendsen velocity rescale thermostat for 0.5 million integration steps for each 2 fs (in total 1 ns). Consequently, the NPT ensemble was used for system equilibration by execution of the Parrinello-Rahman barostat maintaining the system pressure at 1 bar for 1 ns at 300 K. Three independent MD simulation runs were performed for each system under NPT ensemble for 100 ns.

To ensure stability of the triple mutant (Chit19-C191A/C231S/C286T), we analyzed the root-mean-square deviation (RMSD) of C_α_ atom positions over the full trajectory to the reference structure (time step 0) and the root-mean-square fluctuation (RMSF) of single residue positions from the average reference position over the full trajectory. All analyses were performed using GROMACS tools.

# **References**

Abraham, M. J., Murtola, T., Schulz, R., Páll, S., Smith, J. C., Hess, B., & Lindahl, E. (2015). GROMACS: High performance molecular simulations through multi-level parallelism from laptops to supercomputers. *SoftwareX, 1-2*, 19-25. doi:10.1016/j.softx.2015.06.001

Almagro Armenteros, J. J., Tsirigos, K. D., Sønderby, C. K., Petersen, T. N., Winther, O., Brunak, S., . . . Nielsen, H. (2019). SignalP 5.0 improves signal peptide predictions using deep neural networks. *Nature Biotechnology, 37*(4), 420-423. doi:10.1038/s41587-019-0036-z

Altschul, S. (1997). Gapped BLAST and PSI-BLAST: a new generation of protein database search programs. *Nucleic Acids Research, 25*(17), 3389-3402. doi:10.1093/nar/25.17.3389

Altschul, S. F., Wootton, J. C., Gertz, E. M., Agarwala, R., Morgulis, A., Schaffer, A. A., & Yu, Y.-K. (2005). Protein database searches using compositionally adjusted substitution matrices. *FEBS Journal, 272*(20), 5101-5109. doi:10.1111/j.1742-4658.2005.04945.x

Ashkenazy, H., Abadi, S., Martz, E., Chay, O., Mayrose, I., Pupko, T., & Ben-Tal, N. (2016). ConSurf 2016: an improved methodology to estimate and visualize evolutionary conservation in macromolecules. *Nucleic Acids Research, 44*(W1), W344-W350. doi:10.1093/nar/gkw408

Ashkenazy, H., Erez, E., Martz, E., Pupko, T., & Ben-Tal, N. (2010). ConSurf 2010: calculating evolutionary conservation in sequence and structure of proteins and nucleic acids. *Nucleic Acids Res, 38*(Web Server issue), W529-533. doi:10.1093/nar/gkq399

Berendsen, H. J. C., Grigera, J. R., & Straatsma, T. P. (1987). The missing term in effective pair potentials. *The Journal of Physical Chemistry, 91*(24), 6269-6271. doi:10.1021/j100308a038

Berendsen, H. J. C., Van Der Spoel, D., & Van Drunen, R. (1995). GROMACS: A message-passing parallel molecular dynamics implementation. *Computer Physics Communications, 91*(1-3), 43-56. doi:10.1016/0010-4655(95)00042-e

Bowie, J., Lüthy, R., & Eisenberg, D. (1991). A method to identify protein sequences that fold into a known three-dimensional structure. *Science, 253*(5016), 164-170. doi:10.1126/science.1853201

Crooks, G. E., Hon, G., Chandonia, J., & Brenner, S. E. (2004). WebLogo: A Sequence Logo Generator. *Genome Research, 14*(6), 1188-1190. doi:10.1101/gr.849004

Dolinsky, T. J., Czodrowski, P., Li, H., Nielsen, J. E., Jensen, J. H., Klebe, G., & Baker, N. A. (2007). PDB2PQR: expanding and upgrading automated preparation of biomolecular structures for molecular simulations. *Nucleic Acids Research, 35*(Web Server), W522-W525. doi:10.1093/nar/gkm276

Huet, J. l., Rucktooa, P., Clantin, B., Azarkan, M., Looze, Y., Villeret, V., & Wintjens, R. (2008). X-ray Structure of Papaya Chitinase Reveals the Substrate Binding Mode of Glycosyl Hydrolase Family 19 Chitinases. *Biochemistry, 47*(32), 8283-8291. doi:10.1021/bi800655u

Johnson, M., Zaretskaya, I., Raytselis, Y., Merezhuk, Y., McGinnis, S., & Madden, T. L. (2008). NCBI BLAST: a better web interface. *Nucleic Acids Res, 36*(Web Server issue), W5-9. doi:10.1093/nar/gkn201

Kezuka, Y., Kojima, M., Mizuno, R., Suzuki, K., Watanabe, T., & Nonaka, T. (2010). Structure of full-length class I chitinase from rice revealed by X-ray crystallography and small-angle X-ray scattering. *Proteins: Structure, Function, and Bioinformatics, 78*(10), 2295-2305. doi:10.1002/prot.22742

Krieger, E., Koraimann, G., & Vriend, G. (2002). Increasing the precision of comparative models with YASARA NOVA-a self-parameterizing force field. *Proteins: Structure, Function, and Bioinformatics, 47*(3), 393-402. doi:10.1002/prot.10104

Krieger, E., Nabuurs, S. B., & Vriend, G. (2003). Homology modeling. *Methods Biochem Anal, 44*, 509-523. doi:10.1002/0471721204.ch25

Krieger, E., & Vriend, G. (2014). YASARA View—molecular graphics for all devices—from smartphones to workstations. *Bioinformatics, 30*(20), 2981-2982. doi:10.1093/bioinformatics/btu426

Land, H., & Humble, M. S. (2018). YASARA: A Tool to Obtain Structural Guidance in Biocatalytic Investigations. In U. T. Bornscheuer & M. Höhne (Eds.), *Protein Engineering: Methods and Protocols* (pp. 43-67). New York, NY: Springer New York.

Laskowski, R. A., Macarthur, M. W., Moss, D. S., & Thornton, J. M. (1993). PROCHECK: a program to check the stereochemical quality of protein structures. *Journal of Applied Crystallography, 26*(2), 283-291. doi:10.1107/s0021889892009944

Li, H., Robertson, A. D., & Jensen, J. H. (2005). Very fast empirical prediction and rationalization of protein pKa values. *Proteins: Structure, Function, and Bioinformatics, 61*(4), 704-721. doi:10.1002/prot.20660

Lindorff-Larsen, K., Piana, S., Palmo, K., Maragakis, P., Klepeis, J. L., Dror, R. O., & Shaw, D. E. (2010). Improved side-chain torsion potentials for the Amber ff99SB protein force field. *Proteins: Structure, Function, and Bioinformatics, 78*(8), 1950-1958. doi:10.1002/prot.22711

Lüthy, R., Bowie, J. U., & Eisenberg, D. (1992). Assessment of protein models with three-dimensional profiles. *Nature, 356*(6364), 83-85. doi:10.1038/356083a0

Mihalek, I., Reš, I., & Lichtarge, O. (2004). A Family of Evolution–Entropy Hybrid Methods for Ranking Protein Residues by Importance. *Journal of Molecular Biology, 336*(5), 1265-1282. doi:10.1016/j.jmb.2003.12.078

Schymkowitz, J., Borg, J., Stricher, F., Nys, R., Rousseau, F., & Serrano, L. (2005). The FoldX web server: an online force field. *Nucleic Acids Research, 33*(Web Server), W382-W388. doi:10.1093/nar/gki387

Tatusova, T. A., & Madden, T. L. (1999). BLAST 2 Sequences, a new tool for comparing protein and nucleotide sequences. *FEMS Microbiol Lett, 174*(2), 247-250. doi:10.1111/j.1574-6968.1999.tb13575.x

Van Der Spoel, D., Lindahl, E., Hess, B., Groenhof, G., Mark, A. E., & Berendsen, H. J. C. (2005). GROMACS: Fast, flexible, and free. *Journal of Computational Chemistry, 26*(16), 1701-1718. doi:10.1002/jcc.20291

Van Durme, J., Delgado, J., Stricher, F., Serrano, L., Schymkowitz, J., & Rousseau, F. (2011). A graphical interface for the FoldX forcefield. *Bioinformatics, 27*(12), 1711-1712. doi:10.1093/bioinformatics/btr254

Wang, G., & Dunbrack, R. L. (2003). PISCES: a protein sequence culling server. *Bioinformatics, 19*(12), 1589-1591. doi:10.1093/bioinformatics/btg224

Wilkins, A., Erdin, S., Lua, R., & Lichtarge, O. (2012). Evolutionary Trace for Prediction and Redesign of Protein Functional Sites. In (pp. 29-42): Springer New York.
